# Photocatalyzed Aerobic Oxidation of Thiols to Disulfides Using Cu_2_O Polyhedra

**DOI:** 10.1021/acsami.4c21206

**Published:** 2025-03-13

**Authors:** Wan-Ting Dai, Chun-Chia Wen, Hsi-Jui Lin, Michael H. Huang

**Affiliations:** Department of Chemistry, National Tsing Hua University, Hsinchu 300044, Taiwan

**Keywords:** cuprous oxide, facet-dependent properties, photocatalysis, semiconductor, thiol oxidation

## Abstract

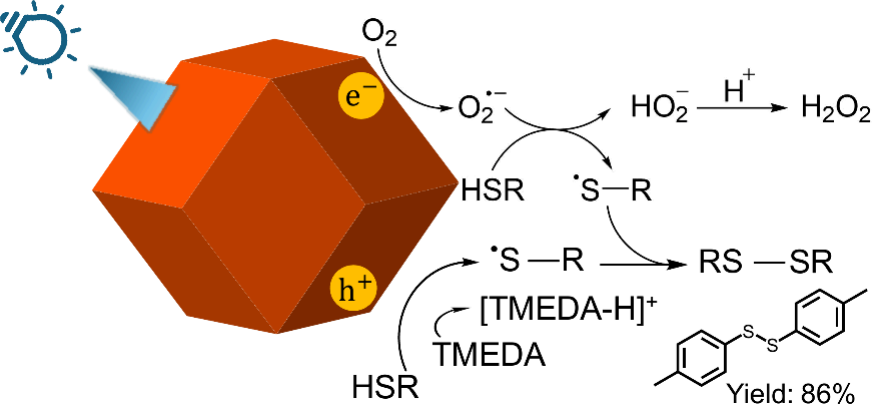

To further demonstrate semiconductor facet effect to photocatalytic organic transformations and address a lack of using simple polyhedral semiconductor particles for disulfide bond formation, Cu_2_O cubes, octahedra, and rhombic dodecahedra were used to photocatalyze aerobic oxidation of 4-methylbenzenethiol. After reaction condition optimization, Cu_2_O crystals and *N*,*N*,*N*′,*N*′-tetramethylethylenediamine (TMEDA) were added to 4-methylbenzenethiol in ethanol for thiol oxidation to 1,2-di-*p*-tolyldisulfane under 390 nm light-emitting diode (LED) lamp irradiation for just 5 min. Rhombic dodecahedra gave the highest product yield, followed by octahedra, cubes, and commercial Cu_2_O powder. Cu_2_O rhombic dodecahedra were subsequently employed to photocatalyze thiols bearing a diverse scope of substituents with satisfactory yields. Reactive species trapping experiments were performed to support a plausible reaction mechanism. Semiconductor crystals with surface control can be a simple but highly effective strategy for enhancing photocatalytic organic transformations.

## Introduction

Various properties of semiconductor crystals exhibit facet dependence, including electrical conductivity, photocatalytic activity, light absorption and emission, magnetism, and piezoelectricity.^1−10^ For example, {110}-bound Cu_2_O rhombic dodecahedra are more photocatalytically active than {111}-terminated octahedra, yet {100}-exposed cubes are inert due to a lack of radical generation upon photoirradiation.^11^ The emergence of all these phenomena can be understood to arise from the presence of a facet-specific surface layer with some lattice deviations, as demonstrated by synchrotron X-ray diffraction (XRD) analysis and high-resolution transmission electron microscopy characterization.^7,9,12^ Moreover, changing the crystal shape can also tune the bulk and surface layer lattice constants. These lattice features arise naturally as the crystals are synthesized, since changes in the reaction quotient *Q* in making particles of different shapes mean that *ΔG*, and hence *ΔS*, should vary.^2^ The entropy difference is manifested in the lattice deviations. Charge transfer is affected if the surface lattice is perturbed, yielding the observed photocatalytic facet dependence. To enhance photocatalytic activity, surface functionalization with conjugated molecules such as 4-nitrophenylacetylene (4-NA) and 4-cyanophenylacetylene has been found to be highly effective.^13,14^ This is because the surface band structure and the electron density has been greatly tuned with the molecular decoration.

Light illumination on semiconductor materials generates photoexcited electrons in the conduction band and holes in the valence band, which then migrate to the surface. Electrons reduce dissolved oxygen forming superoxide anion radicals (O_2_^•–^), while holes oxidize water or hydroxyl ions to produce hydroxyl radicals (^•^OH).^15,16^ Although the charge carriers and radical species have been employed for dye degradation and carbon dioxide reduction, they can also be utilized to catalyze some organic reactions.^17−19^ Previously, Cu_2_O crystals have been used to photocatalyze arylboronic acid hydroxylation.^3,16^ 4-NA-functionalized Cu_2_O crystals can also deliver photoassisted aryl sulfide oxidation, oxidative amine coupling, and dimethylacrylamide polymerization.^4,20,21^ Another reaction that can be explored using Cu_2_O polyhedra is photocatalytic aerobic thiol oxidation to a disulfide. Disulfide bonds are found inβ-sheet secondary structures to stabilize folding conformation of protein.^22,23^ Some bioactive compounds such as Kotamide E with antitumor and anti-inflammatory effects also contain disulfides.^24,25^ Previously, disulfide bonds have generally been achieved using catalysts such as metal complexes, metal/metal oxide particles, and metal–organic frameworks.^26−29^ More recently, photocatalytic oxidation of thiols to form disulfides have been demonstrated using a dye molecule with a complicated oxygen supply system, while use of TEMPO can yield asymmetric disulfides after 2–4.5 h of photoirradiation.^30,31^ Composite nanoparticles deposited on reduced graphene oxide can also photocatalyze thiol oxidation, but the conversion percentages were relatively low.^32^ We consider using simple Cu_2_O polyhedra to photocatalyze this reaction and demonstrate facet effect. Here Cu_2_O cubes, octahedra, and rhombic dodecahedra were employed for photocatalytic aerobic oxidation of 4-methylbenzenethiol, showing rhombic dodecahedra can deliver the highest yield to 1,2-di-*p*-tolyldisulfane in just 5 min. After solvent optimization, rhombic dodecahedra show generally good conversion percentages toward a broad range of thiols. Electron, hole, and radical scavenging tests provide support for the proposed reaction mechanism.

## Experimental Section

### Chemicals

Sodium dodecyl sulfate (SDS, ≥ 99%, J. T. Baker), copper(II) chloride (CuCl_2_, 98%, Alfa Aesar), sodium hydroxide (NaOH, ≥ 98%, Honeywell), hydroxylamine hydrochloride (NH_2_OH·HCl, 99%, Thermo Scientific), copper(I) oxide (Cu_2_O, 97%, Sigma-Aldrich), 4-methylbenzenethiol (CH_3_C_6_H_4_SH, 99%, TCI), thiophenol (C_6_H_5_SH, 97+%, Thermo Scientific), 4-*tert*-butylbenzenethiol ((CH_3_)_3_CC_6_H_4_SH, 97%, Nova-Matls), 4-methoxythiophenol (CH_3_OC_6_H_4_SH, 98%, Nova-Matls), 3-methoxythiophenol (CH_3_OC_6_H_4_SH, 98%, Nova-Matls), 2-methoxythiophenol (CH_3_OC_6_H_4_SH, 98%, Nova-Matls), 4-fluorothiophenol (FC_6_H_4_SH, 98%, Nova-Matls), 4-chlorothiophenol (ClC_6_H_4_SH, 98%, Nova-Matls), 4-bromothiophenol (BrC_6_H_4_SH, 98%, Nova-Matls), 4-(trifluoromethyl)benzenethiol (C_7_H_5_F_3_S, 97%, Nova-Matls), N,N,*N*′,*N*′-tetramethylethylenediamine (TMEDA, (CH_3_)_2_NCH_2_CH_2_N(CH_3_)_2_, 99%, Thermo Scientific), 2-mercaptopyridine (C_5_H_5_NS, 98%, Nova-Matls), 2-naphthalenethiol (C_10_H_7_SH, 98%, Nova-Matls), triphenylmethane (TPM, (C_6_H_5_)_3_CH, 98%, Nova-Matls), chloroform-d (CDCl_3_, 99.8%, Sigma-Aldrich), methanol (CH_3_OH, 99.8%, Honeywell), ethanol (C_2_H_5_OH, 100%, Honeywell), tetrahydrofuran (THF, C_4_H_8_O, 99.8+%, Skysoltech), dimethylformamide (DMF, HCON(CH_3_)_2_, 99.5%, Merck), acetonitrile (ACN, CH_3_CN, 99.5%, J. T. Baker), dichloromethane (DCM, CH_2_Cl_2_, 99%, Honeywell), ethyl acetate (CH_3_COOC_2_H_5_, 99.6%, Macron), hexane (CH_3_(CH_2_)_4_CH_3_, 95%, Duksan), tert-butanol ((CH_3_)_3_COH, ≥ 99.3%, Sigma-Aldrich), sodium azide (NaN_3_, ≥ 99.5, Sigma-Aldrich), 1,4-benzoquinone (C_6_H_4_O_2_, 98+%, Thermo Scientific), potassium iodide (KI, ≥ 99.5%, Sigma-Aldrich), potassium bromate (KBrO_3_, 99%, Alfa Aesar), 5,5-dimethyl-1-pyrroline N-oxide (DMPO, C_6_H_11_NO, 98%, Matrix), 2,2,6,6-tetramethylpiperidine 1-oxyl (TEMPO, 2,2,6,6-tetramethylpiperidine 1-oxyl, 98+%, Alfa Aesar) and silical gel (230 mesh, pH 7, Silicycle) were used as received. All solutions were prepared using ultrapure Milli-Q deionized water (18.2 MΩ).

### General Procedure for Cu_2_O-Photocatalyzed Thiol Oxidation

First, 2.9 mg (0.02 mmol) of Cu_2_O rhombic dodecahedra and 0.4 mmol (2.0 equiv) of 4-methylbenzenethiol were loaded into a 15 mL quartz tube, sealed the tube with a rubber stopper, and evacuated for 10 min using a Schlenk line. The tube was backfilled with oxygen for 1 min. This step was repeated three times. Under oxygen atmosphere, 0.4 mmol (2.0 equiv) of TMEDA and 3.0 mL of acetonitrile were introduced. The mixture was sonicated for 30 s. The tube was magnetically stirred at 1000 rpm, while being irradiated with light from a blue LED (40 W, 390 nm) at a distance of 2 cm from the light source. The light power reaching the cell was measured to be 0.51 W/cm^2^. A fan was used to cool the solution to around room temperature. The photocatalytic reaction took just 5 min.

After the reaction, the reaction mixture was transferred to a 15 mL centrifuge tube, washed with ethanol, centrifuged at 10,000 rpm for 3 min to separate the Cu_2_O crystals. The supernatant was collected and concentrated using a rotary evaporator to remove solvent. Thin-layer chromatography was conducted on silica-gel 60 F_254_ plates (Merck). Column chromatography was performed on the solid product using an appropriate stationary phase and solvent system to further purify the product. The purified product was characterized using nuclear magnetic resonance (NMR) spectroscopy to determine its identity and purity. Triphenylmethane (0.2 mmol) was used as an internal standard.

### Instrumentation

XRD patterns were collected using a D2 PHASER diffractometer with Cu *K*α radiation. The instrumental parameters are 1 mm slit, 0.6 mm grating, 30 kV X-ray tube voltage, and 10 mA current. Scanning electron microscopy (SEM) images were obtained using a JEOL JSM-7000F microscope equipped with a detector for energy-dispersive X-ray spectroscopy (EDS). High-resolution X-ray photoelectron spectra (XPS) were collected using a ULVAC-PHI PHI Quantera II spectrometer. Kessil PR160L lamps provided three light wavelengths (370, 390, and 440 nm with a maximum power of 40 W) for the photocatalytic experiments. NMR analysis was performed using a Bruker AVANCE II 400 spectrometer operating at 400 MHz for proton (^1^H) NMR spectra and 100 MHz for carbon (^13^C) NMR spectra. Chemical shifts were referenced to the residual solvent peak of CDCl_3_ at 7.24 ppm (^1^H NMR) and 77.00 ppm (^13^C NMR), respectively. Splitting patterns are defined as singlet (s), doublet (d), triplet (t), and multiplet (m). Coupling constants (*J*) are reported in Hertz. High-resolution mass (HR-MS) spectra were obtained using a JEOL JMS-700 mass spectrometer equipped with an electrospray ionization source. Electron paramagnetic resonance (EPR) spectra were obtained using a Bruker ELEXSYSE 580 CW/pulse spectrometer.

## Results and Discussion

**Figure 1:**
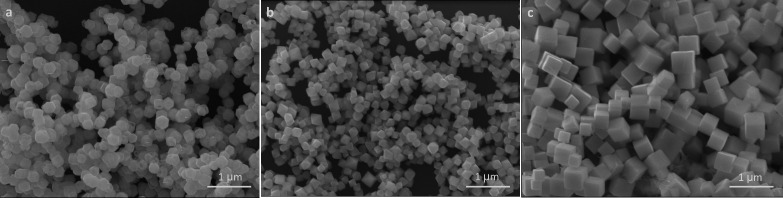
SEM images of the prepared Cu_2_O (a) rhombic dodecahedra, (b) octahedra, and (c) cubes.

Synthesis conditions for Cu_2_O cubes, octahedra, and rhombic dodecahedra are supplied in the Supporting Information. Scheme S1 illustrates the synthesis conditions and the solution color changes. Figure 1 presents SEM images of the synthesized Cu_2_O crystals. The particles are highly uniform in size and shape. Figure S1 provides their size distribution histograms. The average edge length of cubes is 245 nm, while the average opposite corner length of octahedra is 294 nm. For rhombic dodecahedra, their average opposite face length is 445 nm. XRD patterns of these crystals are available in Figure S2. Cubes have a preferentially high (200) peak, while rhombic dodecahedra exhibit stronger (110) and (220) peaks compared to other samples. Interestingly, the samples show peak shifts that match with the reported synchrotron XRD patterns.^12^ For example, the most intense (111) peaks are located at 36.58°, 36.55°, and 36.49° 2θ for cubes, octahedra, and rhombic dodecahedra, respectively. This is the experimental evidence that the lattice constants can vary for different particle shapes to result in diverse facet-dependent behaviors. EDS elemental mapping indicates the presence of copper and oxygen atoms in these crystals with cupper and oxygen atoms in the ratio of roughly 2:1 (Figure S3). EDS line scans of these crystals have been reported.^33^ XPS spectra of Cu_2_O cubes, octahedra, and rhombic dodecahedra are provided in Figure S4. The Cu 2p peak positions indicate only the formation of Cu_2_O.^11^ Satellite peaks from CuO are not observed.

We started with 0.4 mmol of 4-methylbenzenethiol oxidation in ethanol with and without adding Cu_2_O rhombic dodecahedra and in the presence of TMEDA, which acts as a base to remove the thiol group hydrogen (see Table S1). A 390 nm LED lamp at 40 W was used as the light source. Oxygen was pumped into a sealed reaction tube. Interestingly, a longer reaction time is not favorable for disulfide bond formation, so a short reaction time should be used to avoid overoxidation forming sulfenic acids, sulfinic acids, or sulfonic acids. Without adding a catalyst, the yield after 5 min of reaction was 51%, showing there is a pathway using TMEDA and oxygen alone for thiol oxidation. However, upon introducing 5 mol % of Cu_2_O rhombic dodecahedra (2.9 mg), the yield can increase to 70% after just 5 min of reaction, while lower yields were obtained with longer reaction periods. This is close to the shortest reaction time known for this reaction; reaction time can take hours using other catalysts. This notable yield improvement shows that it is highly effective to introduce the Cu_2_O catalyst. By varying the amount of Cu_2_O rhombic dodecahedra used from 3 to 5 mol %, it was found that 5 mol % of the catalyst can give the highest 1,2-di-*p*-tolyldisulfane yield (Table S2).

Next, control experiments were performed (Table 1). In the absence of Cu_2_O crystals, the yield dropped to 51%. Without adding TMEDA, only a trace amount of product was formed, showing its importance to reactivity. Replacing oxygen with nitrogen resulted in minimal product formation. Without light irradiation, the yield was 40%. These results indicate that the reaction requires a catalyst, a base, oxygen, and a light source. To investigate the influence of light wavelength to reactivity, 370 and 440 nm LED lamps were also used (Table S3). Compared to 70% product yield using a 390 nm LED lamp, irradiation with 370 nm light gave a lower yield of 63%. Use of 440 nm light resulted in a substantial yield decrease to 47%. With a larger power density of 0.73 W/cm^2^ than that of the 390 nm lamp, and similar absorbance values in this region for these Cu_2_O crystals, it is unclear why these lamps gave lower product yields.^21^ The 390 nm LED lamp remains the best light source.

**Table 1:**
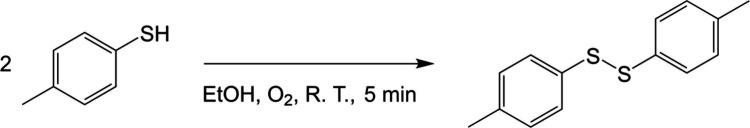
Control Experiments for Thiol Oxidation to Disulfide

entry	Cu_2_O RDs	TMEDA	O_2_	light	yield (%)^a^
1	+	+	+	+	70
2	–	+	+	+	51
3	+	–	+	+	trace
4^b^	+	+	–	+	15
5	+	+	+	–	40

a Reaction conditions: Cu_2_O RDs (2.9 mg), 4-methylbenzenethiol (0.4 mmol), TMEDA (0.4 mmol), ethanol (3.0 mL), 390 nm LED (40 W). Triphenylmethane as an internal standard.

b N_2_ atmosphere.

To compare the reactivity of different Cu_2_O crystals, the total particle surface area should be kept the same. Table S4 gives calculations of the particle weights needed for Cu_2_O cubes, octahedra, and rhombic dodecahedra. Table 2 shows the particle weights. Commercially available Cu_2_O powder having the same weight as that of rhombic dodecahedra was also evaluated. The yield of commercial Cu_2_O powder was only 54%, illustrating the advantage of catalyst surface control to reactivity. Cubes gave a yield of 57%, and 63% for octahedra. Rhombic dodecahedra still are the best catalyst. The facet-dependent photocatalytic activity is present because the barrier or efficiency to electron and hole transport to the particle surfaces is different due to lattice variations within the crystals.^11^ That is why photocatalyzed reactions can best demonstrate semiconductor facet effects. SEM images of the particles after the thiol oxidation reaction indicates preservation of particle morphologies (Figure S5). XRD patterns also show the presence of Cu_2_O with the same peak intensity (Figure S6).

**Table 2:**
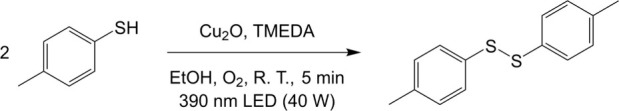
Photocatalytic Activity Comparison of Different Cu_2_O Crystals

entry	catalyst	amount (mg)	yield (%)^a^
1	commercial Cu_2_O	2.9	54
2	Cu_2_O cubes	1.6	57
3	Cu_2_O octahedra	1.1	63
4	Cu_2_O RDs	2.9	70

a Reaction conditions: 4-methylbenzenethiol (0.4 mmol), TMEDA (0.4 mmol), ethanol (3.0 mL). Triphenylmethane as the internal standard.

Next, various solvents were tested for reactivity (Table 3). The use of polar protic solvents, such as water, methanol, and ethanol, resulted in yields of 31%, 63%, and 70%, respectively. Nonpolar solvents, such as THF and DCM, gave 48% and 58% yields, respectively. Interestingly, polar aprotic solvents, DMF and acetonitrile (ACN), exhibited superior performance, achieving yields of 73% and 86%, respectively. Both DMF and ACN have relatively large dielectric constants and dipole moments to possibly stabilize the radical species formed in the reaction, and have better solubility for 4-methylbenzenethiol and 1,2-di-*p*-tolyldisulfane.^34^ These factors contribute to higher product yields. Acetonitrile was the chosen solvent for substrate scope analysis, although ethanol is more environmentally benign.

**Table 3:**
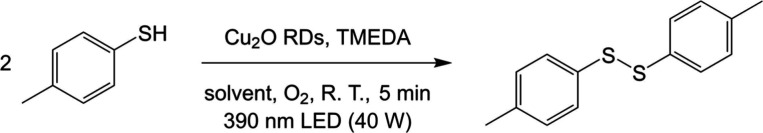
Solvent Optimization Test

entry	solvent	yield (%)^a^
1	H_2_O	31
2	MeOH	63
3	EtOH	70
4	THF	48
5	DCM	58
6	DMF	73
7	ACN	86

a Reaction conditions: Cu_2_O RDs (2.9 mg), 4-methylbenzenethiol (0.4 mmol), TMEDA (0.4 mmol), solvent (3 mL). Triphenylmethane as an internal standard.

Under optimal reaction conditions, varying the substituents on the starting material resulted in a diverse range of products (see Table 4). Alkyl groups (H, Me, tBu) gave yields of 89%, 86%, and 72%, respectively (Entries 1–3). The electron-donating OMe group showed position-dependent yields *para* (nearly 99%), *meta* (80%), and *ortho* (71%) (Entries 4–6). The location of OMe group can significantly impact the yield. For electron-withdrawing groups (F, Cl, Br, I, and CF_3_), the yields were 87%, 83%, 86%, and 71%, respectively (Entries 7–10). Finally, for heterocyclic compounds 2-mercaptopyridine and 2-naphthalenethiol, the yields were 62% and 13%, respectively (Entries 11–12). Low yields are generally the case for aerobic oxidation of heterocyclic compounds. Clearly, Cu_2_O rhombic dodecahedra can photocatalyze thiol oxidation with compounds bearing a diverse range of substituents.

**Table 4:**
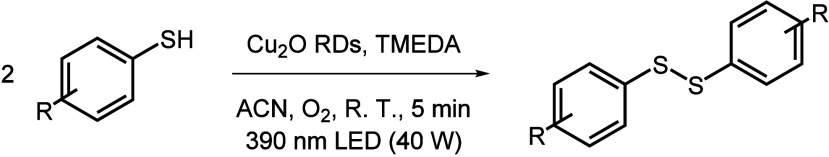

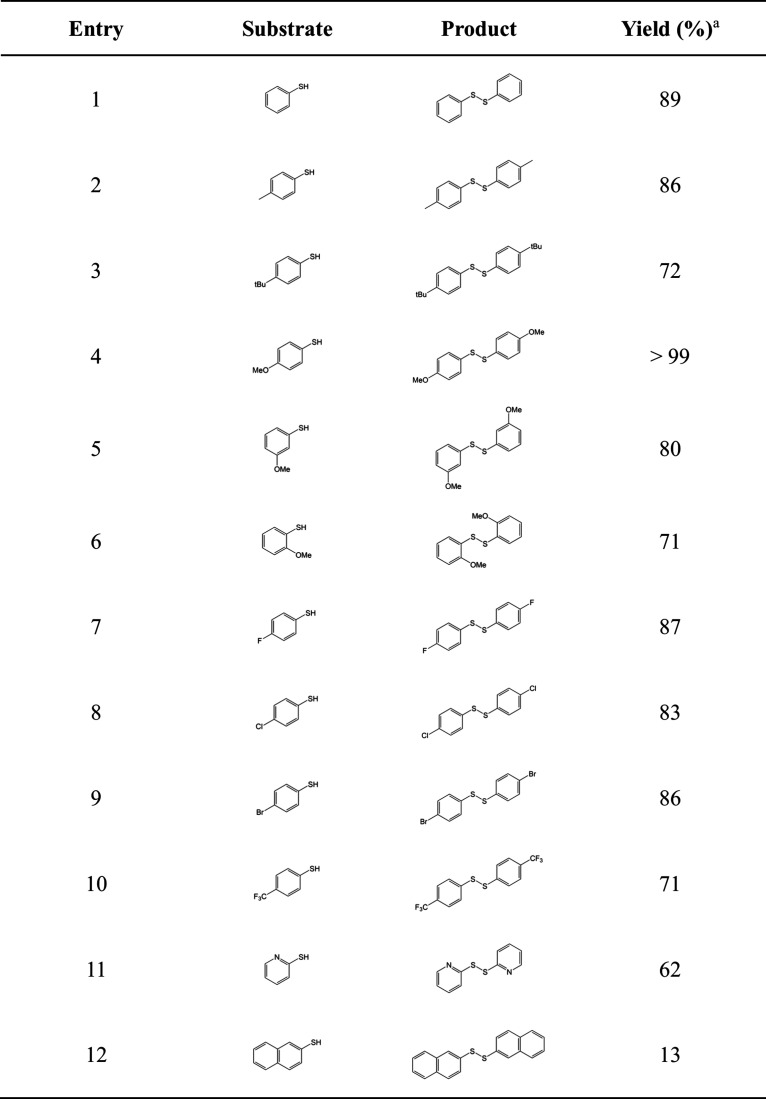
Substrate Scope

a Reaction conditions: Cu_2_O RDs (2.9 mg), thiol (0.4 mmol), TMEDA (0.4 mmol), ACN (3 mL). Triphenylmethane as an internal standard.

To understand the aerobic oxidation mechanism of thiols to disulfides, a series of active species trapping experiments were performed. Table 5 summarizes the results. The product yield was reduced from 86% to 68% and 60% in the presence of KI, a hole scavenger, and KBrO_3_, an electron scavenger, respectively. Expectedly, both photoexcited electrons and holes should participate in the oxidation reaction. Addition of a common hydroxyl radical scavenger, *tert*-butanol, resulted in a slight decrease in yield to 79%, showing hydroxyl radicals are not important to this reaction. A significant decrease in yield to 46% was observed upon the introduction of 1,4-benzoquinone, a superoxide scavenger. Superoxide anion radicals should be actively involved in the oxidation reaction. A similar result was obtained when NaN_3_, a singlet oxygen scavenger, was added. Singlet oxygen can take part in benzylamine coupling reactions.^5^ Singlet oxygen is generated through photoexcition of the ground state triplet oxygen, and is subsequently reduced to give a superoxide anion radical. Removal of singlet oxygen molecules thereby lowers the superoxide radical amount.

**Table 5:**
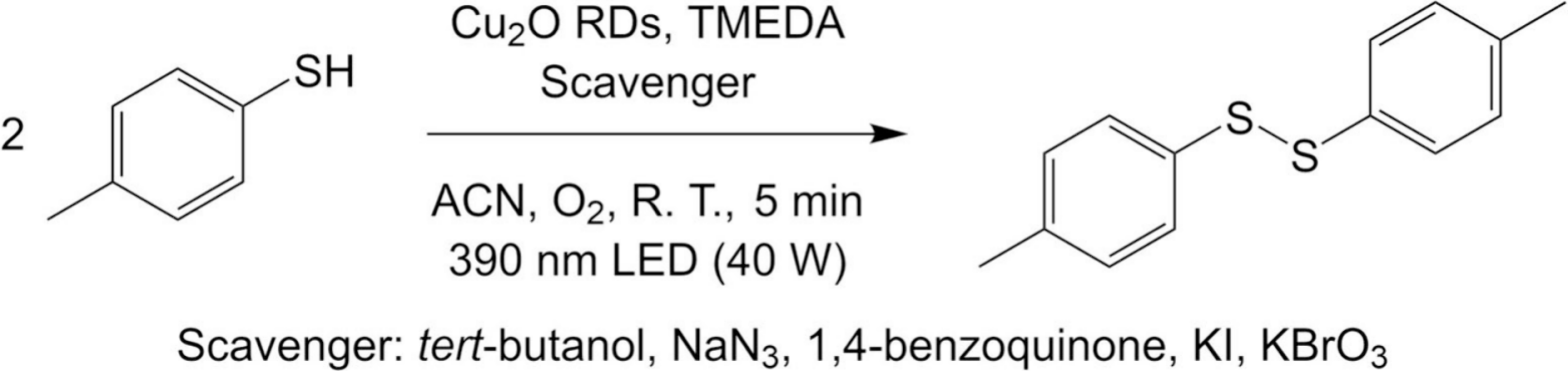
Active Species Trapping Experiments

entry	scavenger (1.0 equiv)	inhibited species	yield (%)^a^
1	KI	h^+^	68
2	KBrO_3_	e^–^	60
3	*tert*-butanol	•OH	79
4	1,4-benzoquinone	O_2_^•–^	46
5	NaN_3_	^1^O_2_	45

a Reaction conditions: Cu_2_O RDs (2.9 mg), 4-methylbenzenethiol (0.4 mmol), TMEDA (0.4 mmol), ACN (3 mL). Triphenylmethane as an internal standard.

Next, we utilized electron paramagnetic resonance spectra to verify the formation of reactive oxygen species. Due to the typically short lifespan of free radicals, DMPO was employed as a spin trapping agent to form relatively stable radical adducts upon reaction with free radicals. When DMPO captures a superoxide radical, it forms a DMPO-OOH adduct. DMPO-OOH adducts are identified by their unique 12-line EPR spectrum.^35^ Due to their short lifespan, DMPO-OOH adducts readily decompose into DMPO–OH adducts, displaying a 4-line spectrum with a relative intensity ratio of 1:2:2:1. When methanol is the solvent, the EPR spectrum displays six peaks (Figure S7). This indicates that superoxide radicals are formed upon light irradiation. Moreover, the mechanism of aerobic thiol oxidation should involve the formation of thiyl radicals (^•^SR).^36,37^ The intermediary thiyl radical can be probed by using TEMPO to capture the radical, and then taking high-resolution mass spectrometry (HR-MS) of the adduct. Under standard conditions with 4-methylbenzenethiol, the addition of 2.0 equiv of TEMPO resulted in the formation of TEMPO-trapped thiyl radical (Figure S8). HR-MS data reveal the presence of TEMPO-trapped thiyl radical.

**Figure 2:**
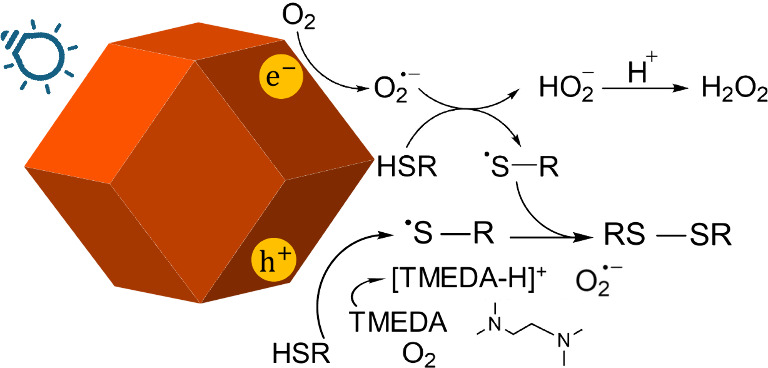
Proposed reaction mechanism for Cu_2_O-photocatalyzed thiol oxidation.

From the above experiments, a plausible reaction mechanism is presented in Figure 2. Under light irradiation, TMEDA molecules react with thiol molecules (RSH) to form thiyl radicals (RS^•^) while also donating electrons to oxygen to generate superoxide radicals. A coupling reaction between thiyl radicals occurs to form disulfide molecules. In the presence of Cu_2_O rhombic dodecahedra, another pathway to enhance the disulfide yield is operative. With light illumination, electron–hole pairs are generated and migrate to the crystal surface. The photoexcited electrons reduce oxygen molecules to form superoxide radicals. Simultaneously, holes react with thiol molecules (HSR), causing them to lose a proton (H^+^) and form thiyl radicals (RS^•^). Another molecule of HSR reacts with a superoxide anion radical, producing another thiyl radical and HO_2_^^–^^. The unstable HO_2_^^–^^ reacts with a proton to form hydrogen peroxide. Coupling of thiyl radicals yield the disulfide product. This additional pathway from Cu_2_O crystals improves the overall disulfide yield.

## Conclusions

Cu_2_O cubes, octahedra, and rhombic dodecahedra were employed for photocatalytic aerobic oxidation of 4-methylbenezenethiol to 1,2-di-*p*-tolyldisulfane. Slight XRD peak shifts in these crystals confirm their lattice constant variations to give various facet-dependent behaviors. In ethanol, Cu_2_O rhombic dodecahedra give the highest yield after just 5 min of light illumination, followed by octahedra, cubes, and commercial available Cu_2_O powder. In acetonitrile, Cu_2_O rhombic dodecahedra have been used to photocatalyze a diverse scope of thiols forming the corresponding disulfides with generally high yields. This work demonstrates again that semiconductor surface control can be highly effective to boosting organic coupling reaction yields, because certain crystal faces can facilitate more efficient charge transfer.

## Supporting Information Available

Size distribution histograms, XRD patterns, particle amount calculations, additional photocatalysis results, SEM images, EPR spectrum, mass spectrum, and NMR spectra and peak positions (PDF)
