# Supplementary material for: Photocatalyzed Aerobic Oxidation of Thiols to Disulfides Using Cu2O Polyhedra
Source: ACS Appl Mater Interfaces. 2025 Mar 13;17(12):18268–74. doi: 10.1021/acsami.4c21206 (PMC11955942; doi:10.1021/acsami.4c21206)
Supplement: Supplementary file 1 — am4c21206_si_001.pdf [file am4c21206_si_001.pdf]

Supporting Information

Photocatalyzed Aerobic Oxidation of Thiols to Disulfides Using Cu_2O Polyhedra

Wan-Ting Dai, Chun-Chia Wen, Hsi-Jui Lin, and Michael H. Huang*

Department of Chemistry, National Tsing Hua University, Hsinchu 300044, Taiwan

E-mail: hyhuang@mx.nthu.edu.tw

Synthesis of Cu_2O Cubes, Octahedra, and Rhombic Dodecahedra. First, 0.3480 g (1.2 mmol) of SDS and deionized water (38.20, 26.20, and 27.68 mL for making cubes, octahedra, and rhombic dodecahedra, respectively) were loaded into a 60 mL vial. After stirring the mixture for 10 min, the vial was kept in a 31 ºC water bath. Next, 0.1 M $CuCl_2$ solution (0.4, 0.8, and 2.0 mL for cubes, octahedra, and rhombic dodecahedra, respectively) was introduced and stirred for 20 min. Following this, 1.0 M NaOH solution (0.80 mL for making cubes and octahedral, and 0.72 mL for growing rhombic dodecahedra) was introduced and stirred for 4 sec (5 sec for octahedra). For cubes and octahedra, 0.6 and 2.6 mL of 0.2 M $NH_2OH·HCl$ solution was then quickly added and stirred for 20 and 10 sec, respectively. For rhombic dodecahedra, 9.6 mL of 0.1 M $NH_2OH·HCl$ solution was quickly added and stirred for 20 sec. The reaction mixture was left undisturbed for 50 min for cubes and rhombic dodecahedra, and 25 min for octahedra.

Following the aging period, the solution was centrifuged at 10000 rpm for 3 min. The Cu_2O particles were thoroughly washed three times to remove unreacted chemicals and residual SDS surfactant using a 1:1 volume ratio of deionized water and ethanol, followed by a final rinse with absolute alcohol. The particles were dried and stored in a vacuum oven for optimal preservation.

Trapping Species Experiment. 2.9 mg (0.02 mmol) of Cu_2O rhombic dodecahedra, 0.4 mmol (2.0 eq.) of 4-methylbenzenethiol, and 0.2 mmol (1.0 eq.) of scavenger were loaded into a 15 mL quartz tube. The tube was sealed with a rubber stopper, evacuated for 10 min using a Schlenk line, and backfilled with oxygen for 1 min. This step was repeated three times. Under oxygen atmosphere, 0.4 mmol (2.0 eq.) of TMEDA and 3.0 mL of ethanol were added. The rest of the procedure is the same as that of the standard photocatalysis reaction.

Electron Paramagnetic Resonance Experiment. 34 mg of DMPO was dissolved in 3 mL of methanol. Activated carbon was added to the solution and sonicated for 1 min to remove DMPO impurities. The mixture was centrifuged to remove the activated carbon and obtain a pure DMPO solution. In air atmosphere, 2.9 mg of Cu_2O RDs and DMPO solution were loaded into a 4 mL vial. The mixture was sonicated for 1 min to ensure homogeneous dispersion of the nanoparticles. The mixture was irradiated with light from a 500 W xenon light (λ_{max} > 400 nm) for 2 min

to generate free radicals. Immediately after irradiation, the mixture was transferred to an aluminum foil-covered 1.5 mL Eppendorf tube, and promptly transported to the EPR instrument room for analysis.

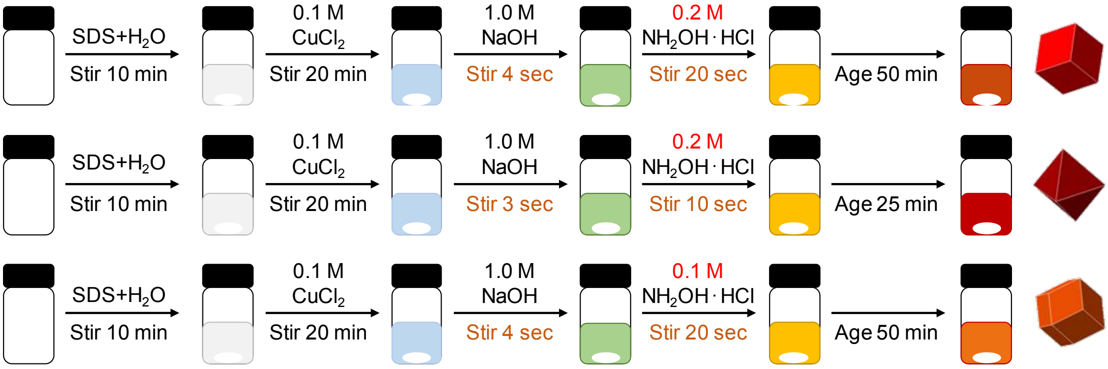

Scheme S1. Cu_2O crystal synthesis procedures and the solution and the solution color changes.

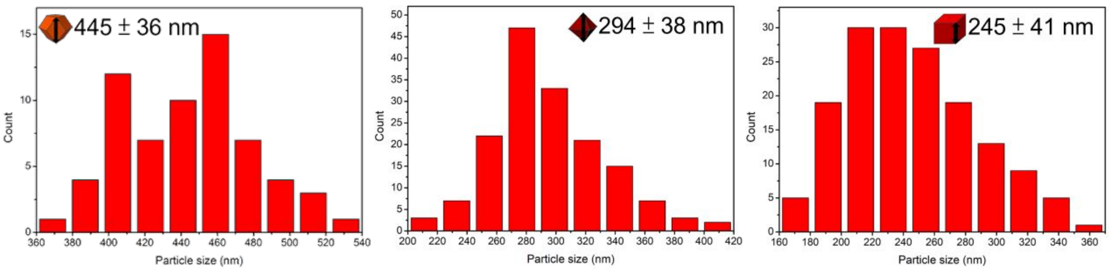

Figure S1. Size distribution histograms of the prepared Cu_2O polyhedra. The models indicate the measured particle sizes.

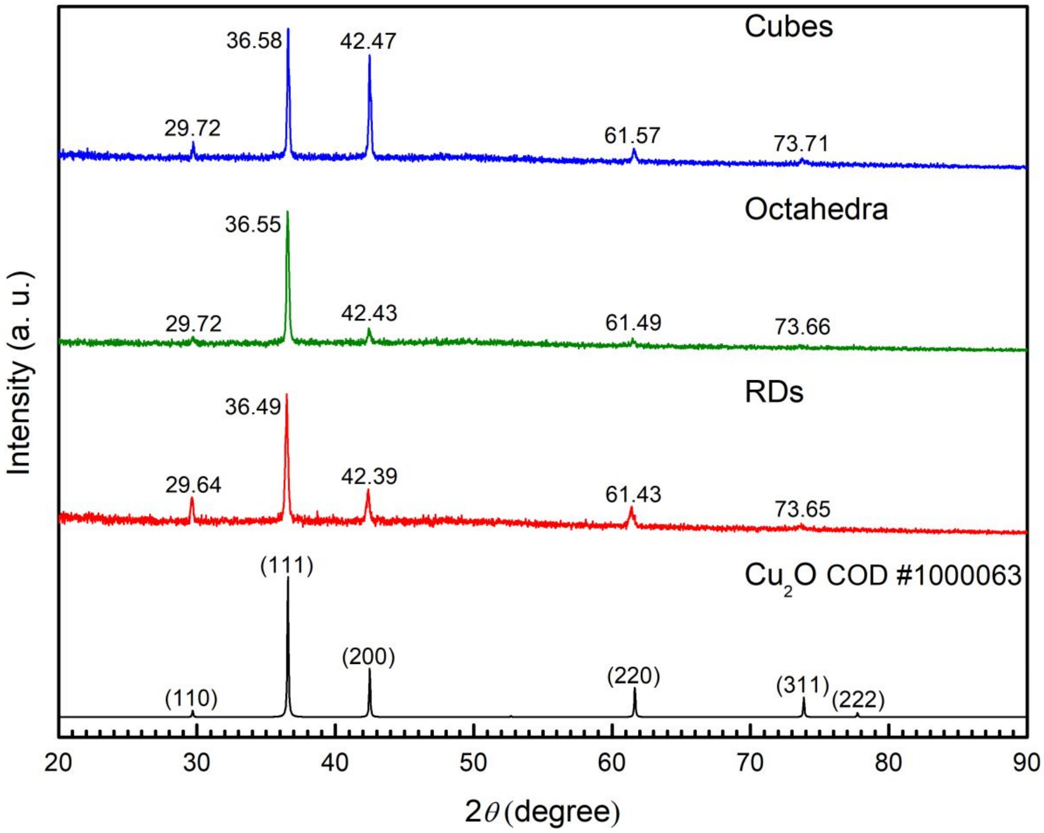

Figure S2. XRD patterns of the synthesized Cu_2O polyhedra and a reference pattern.

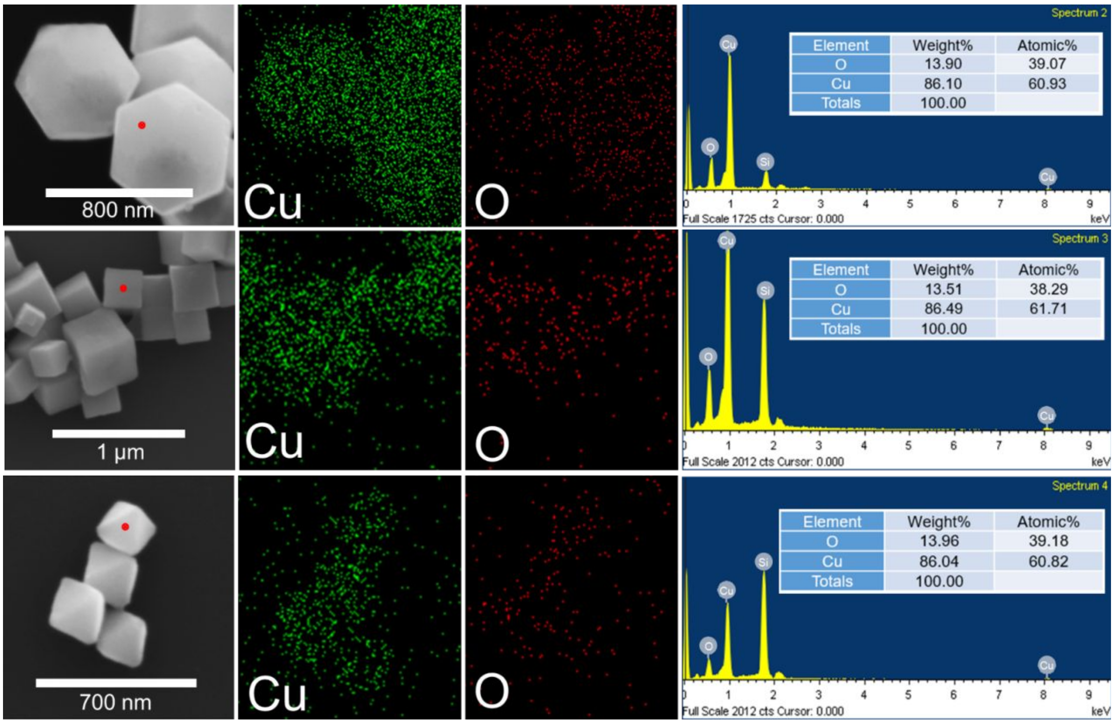

Figure S3. EDS spectra and elemental mapping of Cu_2O rhombic dodecahedra, cubes, and octahedra. The Si signal comes from the silicon substrate used to load Cu_2O particles.

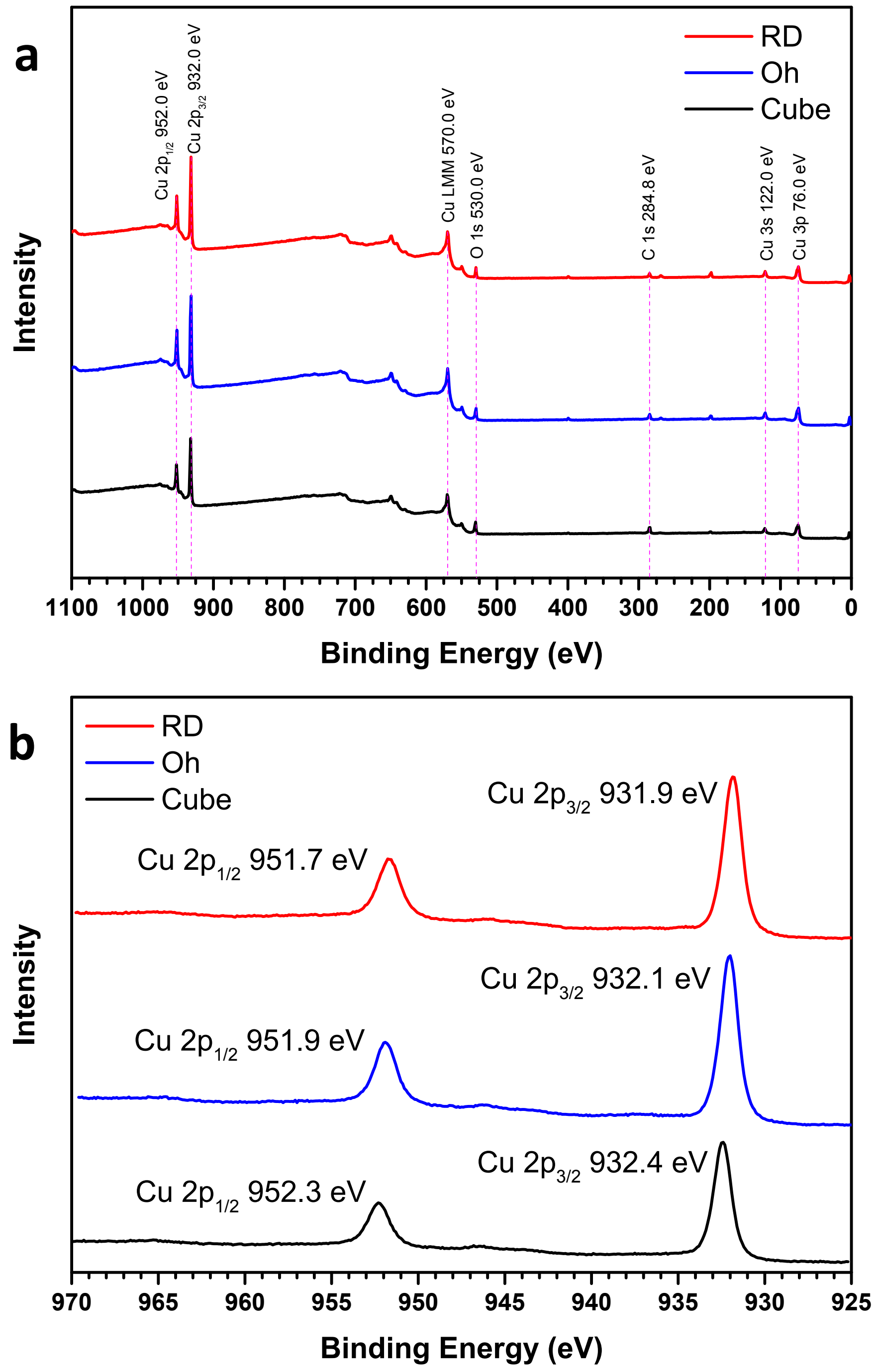

Figure S4. (a) Full XPS spectra of the Cu_2O cubes, octahedra, and rhombic dodecahedra. (b) Expanded spectra showing the Cu 2p peaks.

Table S1. Reaction Time Effect on Product Yields

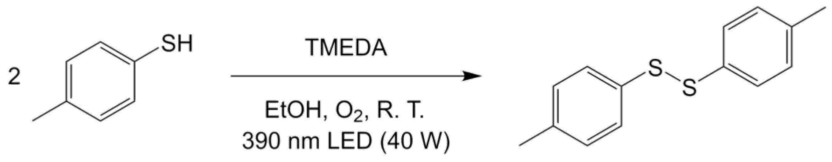

Entry	Catalyst	Time (min)	Yield (%)[a]
1	–	20	44
2	–	5	51
3	Cu_2O RDs	20	50
4	Cu_2O RDs	15	62
5	Cu_2O RDs	10	67
6	Cu_2O RDs	5	70
7	Cu_2O RDs	2	43

Reaction conditions: Cu_2O RDs (2.9 mg), 4-methylbenzenethiol (0.4 mmol), TMEDA (0.4 mmol), ethanol (3.0 mL). [a]Triphenylmethane as an internal standard.

Table S2. Catalyst Amount Effect

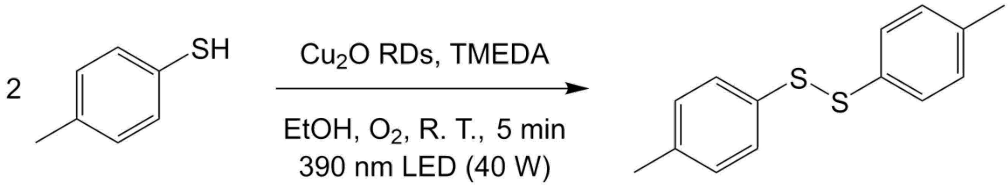

Entry	Catalyst	Yield (%)[a]
1	Cu_2O RDs (3 mol %)	54
2	Cu_2O RDs (4 mol %)	63
3	Cu_2O RDs (5 mol %)	70

Reaction conditions: 4-methylbenzenethiol (0.4 mmol), TMEDA (0.4 mmol), ethanol (3.0 mL). [a]Triphenylmethane as an internal standard.

Table S3. Light Wavelength Effect

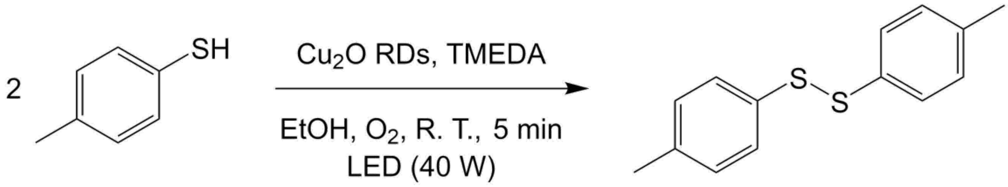

Entry	Wavelength (nm)	Yield (%)[a]
1	370	63
2	390	70
3	440	47

Reaction conditions: Cu_2O RDs (2.9 mg), 4-methylbenzenethiol (0.4 mmol), TMEDA (0.4 mmol), ethanol (3.0 mL). [a]Triphenylmethane as an internal standard.

Table S4. Calculations of the Particle Weights Needed for Photocatalysis Reaction

	cubes	octahedra	RDs
size (nm)	245	294	445
surface area of a single particle (nm^2)	3.60×10^5	1.50×10^5	8.40×10^5
volume of a single particle (nm^3)	1.47×10^7	4.24×10^6	6.23×10^7
density of Cu_2O (mg/nm^3)		6.0×10^{-18}	
weight of a single particle (mg)	8.82×10^{-11}	2.54×10^{-11}	3.74×10^{-10}
fixed surface area (nm^2)		6.5×10^{15}	
number of particles	1.81×10^{10}	4.33×10^{10}	7.74×10^9
weight (mg)	1.6	1.1	2.9

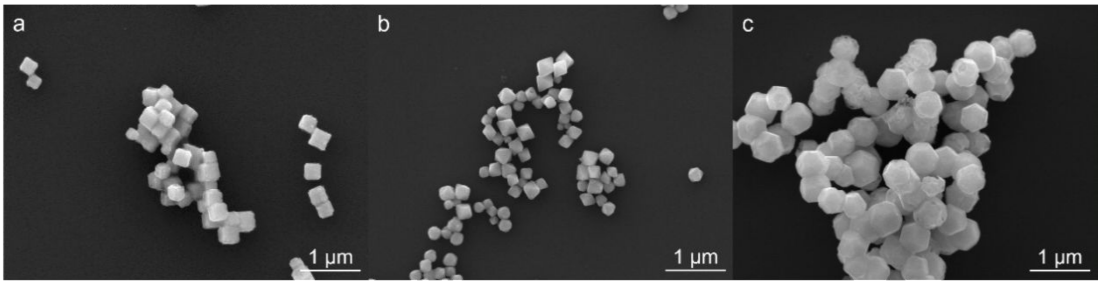

Figure S5. SEM images of the Cu_2O (a) cubes, (b) octahedra, and (c) rhombic dodecahedra after the photocatalysis reaction.

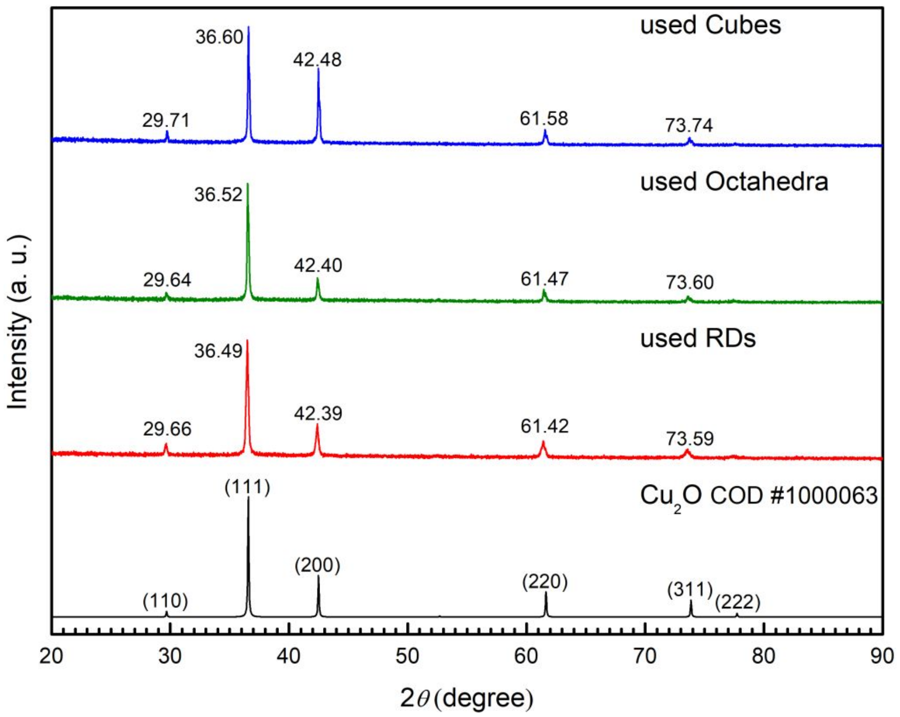

Figure S6. XRD patterns of the Cu_2O polyhedra after the photocatalysis reaction.

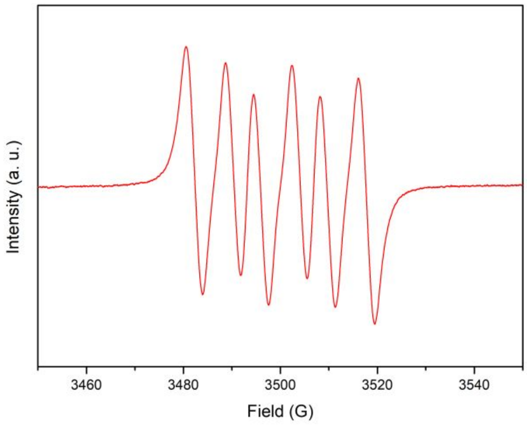

Figure S7. EPR spectrum of photoirradiated Cu_2O rhombic dodecahedra measured in methanol.

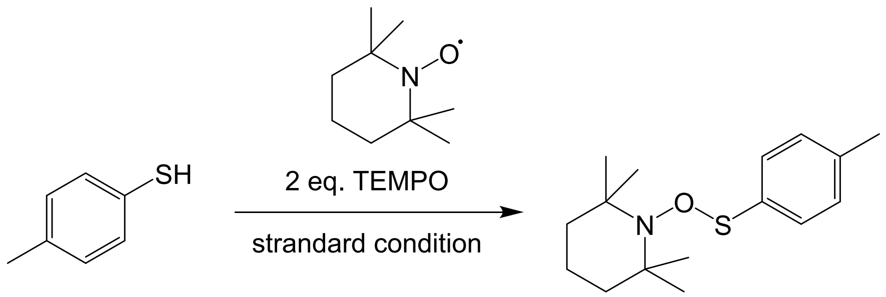

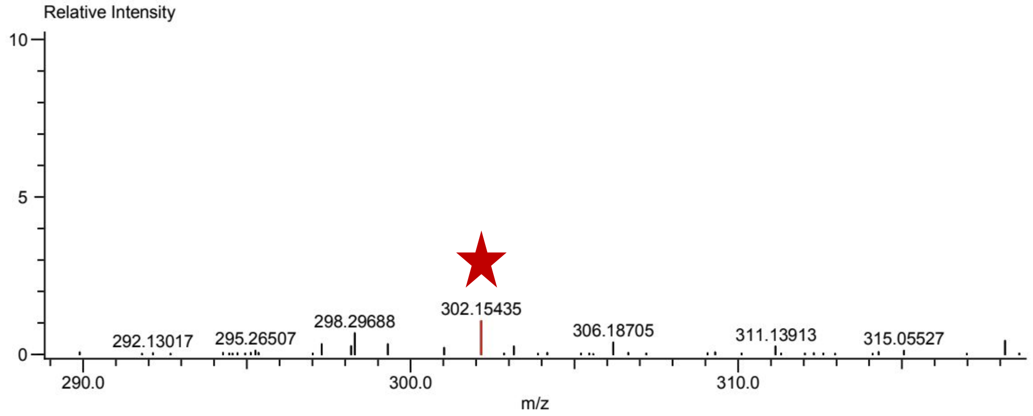

Mass	Intensity	Calc. Mass	Mass Difference [mDa]	Mass Difference [ppm]	Possible Formula
302.15435	3965.48	302.15545	-1.10	-3.64	$^{12}C_{16}{}^{1}H_{25}{}^{14}N_{1}{}^{23}Na_{1}{}^{16}O_{1}{}^{32}S_{1}$

Figure S8. Free radical trapping of 4-methylbenzenethiol with TEMPO. HR-MS (ESI) m/z calculated for $C_{16}H_{25}NOSNa^{+}$: 302.1555; found: 302.1544.

NMR Spectroscopic Data

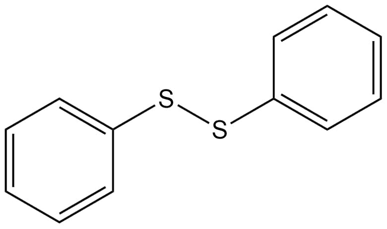

1,2-diphenyldisulfane

^{1}H NMR (400 MHz, $CDCl_3$): δ = 7.51 (d, *J* = 7.5 Hz, 4H; Ar−H), 7.31 (t, *J* = 7.4 Hz, 4H; Ar−H), 7.25-7.21 (m, 2H; Ar−H); ^{13}C NMR (100 MHz, $CDCl_3$): δ = 136.98 (C), 129.01 (CH), 127.47 (CH), 127.11 (CH).

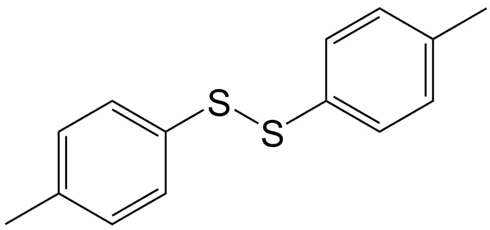

1,2-di-*p*-tolyldisulfane

^{1}H NMR (400 MHz, $CDCl_3$): δ = 7.39 (d, *J* = 8.2 Hz, 4H; Ar−H), 7.10 (d, *J* = 8.0 Hz, 4H; Ar−H), 2.32 (s, 6H; CH_3); ^{13}C NMR (100 MHz, $CDCl_3$): δ = 137.39 (C), 133.85 (C), 129.73 (CH), 128.49 (CH), 21.00 (CH_3).

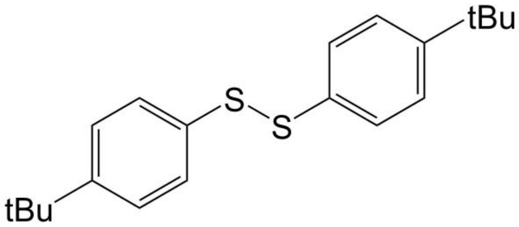

1,2-bis(4-(tert-butyl)phenyl)disulfane

^{1}H NMR (400 MHz, $CDCl_3$): δ = 7.48 (d, *J* = 8.3 Hz, 4H; Ar−H), 7.35 (d, *J* = 8.3 Hz, 4H; Ar−H), 1.33 (s, 18H; CH_3); ^{13}C NMR (100 MHz, $CDCl_3$): δ = 150.42(C), 134.00 (C), 127.70 (CH), 126.07 (CH), 34.49 ($C(CH_3)_3$), 31.24 ($C(CH_3)_3$).

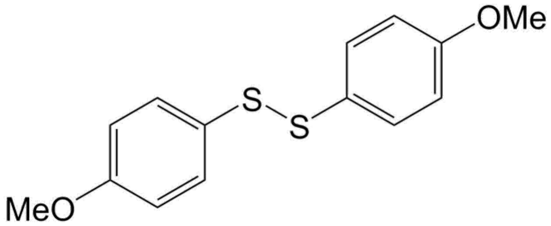

1,2-bis(4-methoxyphenyl)disulfane

^{1}H NMR (400 MHz, $CDCl_3$): δ = 7.38 (d, *J* = 8.0 Hz, 4H; Ar−H), 6.82 (d, *J* = 8.0 Hz,

4H; Ar–H), 3.78 (s, 6H; OCH_3). ^{13}C NMR (100 MHz, $CDCl_3$): δ=159.91 (C), 132.65 (CH), 128.44 (C), 114.61 (CH), 55.36 (OCH_3).

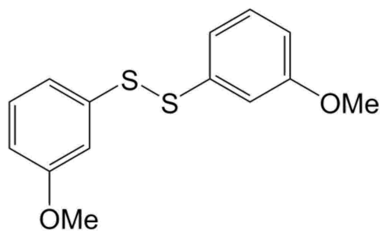

1,2-bis(3-methoxyphenyl)disulfane

^1H NMR (400 MHz, $CDCl_3$): δ = 7.21 (t, J = 8.1 Hz, 2H; Ar–H), 7.10-7.08 (m, 4H; Ar–H), 6.77-6.75 (m, 2H; Ar–H), 3.75 (s, 6H; OCH_3). ^{13}C NMR (100 MHz, $CDCl_3$): δ = 159.97 (C), 138.18 (C), 129.81 (CH), 119.50 (CH), 113.02 (CH), 112.52 (CH), 55.17 (OCH_3).

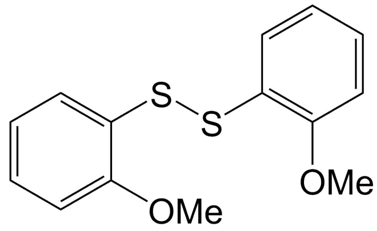

1,2-bis(2-methoxyphenyl)disulfane

^1H NMR (400 MHz, $CDCl_3$): δ = 7.52 (d, J = 7.5 Hz, 2H; Ar–H), 7.17 (t, 2H; Ar–H), 6.89 (t, J = 7.4 Hz, 2H; Ar–H), 6.84 (d, J = 8.0 Hz, 2H; Ar–H), 3.88 (s, 6H; OCH_3). ^{13}C NMR (100 MHz, $CDCl_3$): δ = 156.44 (C), 127.62 (CH), 127.44 (CH), 124.40 (C), 121.17 (CH), 110.37 (CH), 55.73 (OCH_3).

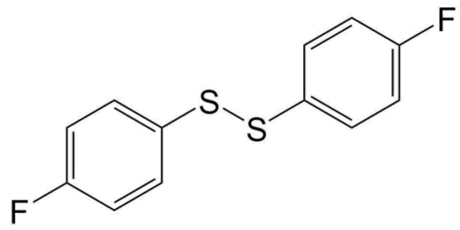

1,2-bis(4-fluorophenyl)disulfane

^1H NMR (400 MHz, $CDCl_3$): δ = 7.43 (dd, J = 8.6, 5.2 Hz, 4H; Ar–H), 76.99 (t, J = 8.6 Hz, 4H; Ar–H) ^{13}C NMR (100 MHz, $CDCl_3$): δ = 162.58 (d, $^1J_{C\text{-}F}$ = 248.2 Hz), 132.15 (d, $^4J_{C\text{-}F}$ = 2.8 Hz), 131.25 (d, $^3J_{C\text{-}F}$ = 8.2 Hz), 116.24 (d, $^2J_{C\text{-}F}$ = 22.2 Hz).

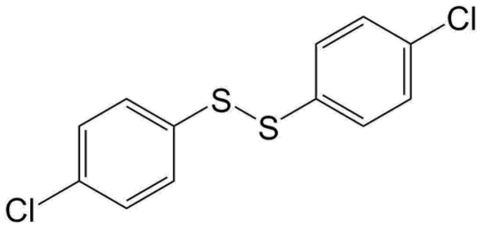

1,2-bis(4-chlorophenyl)disulfane

^{1}H NMR (400 MHz, $CDCl_3$): δ = 7.38 (d, J = 8.3 Hz, 4H; Ar−H), 7.26 (d, J = 8.4 Hz, 4H; Ar−H). ^{13}C NMR (100 MHz, $CDCl_3$): δ = 135.05 (C), 133.55 (C), 129.24 (CH), 129.21 (CH).

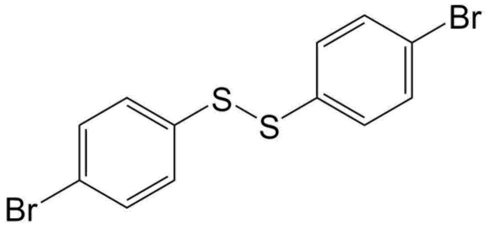

1,2-bis(4-bromophenyl)disulfane

^{1}H NMR (400 MHz, $CDCl_3$): δ = 7.41-7.38 (m, 4H; Ar−H), 7.33-7.30 (m, 4H; Ar−H); ^{13}C NMR (100 MHz, $CDCl_3$): δ = 135.64 (C), 132.13 (CH), 129.30 (CH), 121.46 (C).

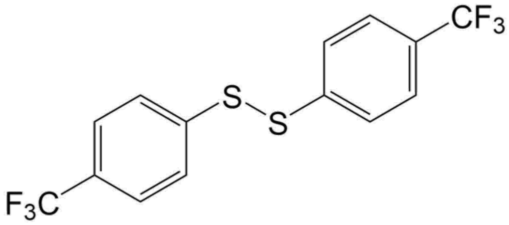

1,2-bis(4-(trifluoromethyl)phenyl)disulfane

^{1}H NMR (400 MHz, $CDCl_3$): δ = 756 (s, 8H; Ar−H); ^{13}C NMR (100 MHz, $CDCl_3$): δ = 140.82 (CH), 129.44 (q, $^2J_{C\text{-}F}$ = 329 Hz), 127.90, 126.59 (C), 126.10 (d, $^4J_{C\text{-}F}$ = 3.3 Hz), 125.20 (C), 122.49 (C), 119.79 (C).

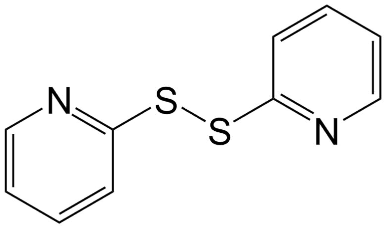

1,2-di(pyridin-2-yl)disulfane

^{1}H NMR (400 MHz, $CDCl_3$): δ = 8.42-7.40 (m, 2H; Ar−H), 7.59-7.52 (m, 4H; Ar−H), 7.07-7.03 (m, 2H; Ar−H); ^{13}C NMR (100 MHz, $CDCl_3$): δ = 158.98 (C), 149.57 (CH), 137.38 (CH), 121.09 (CH), 119.69 (CH).

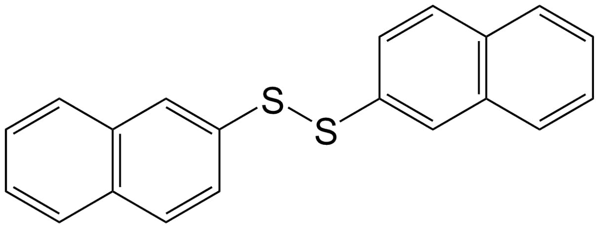

1,2-di(naphthalen-2-yl)disulfane

^{1}H NMR (400 MHz, $CDCl_3$): δ = 7.97 (d, J = 1.8 Hz, 4H; Ar−H), 7.78-7.76 (m, 4H; Ar−H), 7.73-7.70 (m, 2H), 7.60 (dd, J = 8.7, 1.9 Hz, 2H), 7.46-7.42 (m, 4H); ^{13}C NMR (100 MHz, $CDCl_3$): δ = 133.44 (C), 132.47 (C), 128.93 (CH), 127.73 (CH), 127.43 (CH), 126.70 (CH), 126.54 (CH), 126.20 (CH), 125.64 (CH).

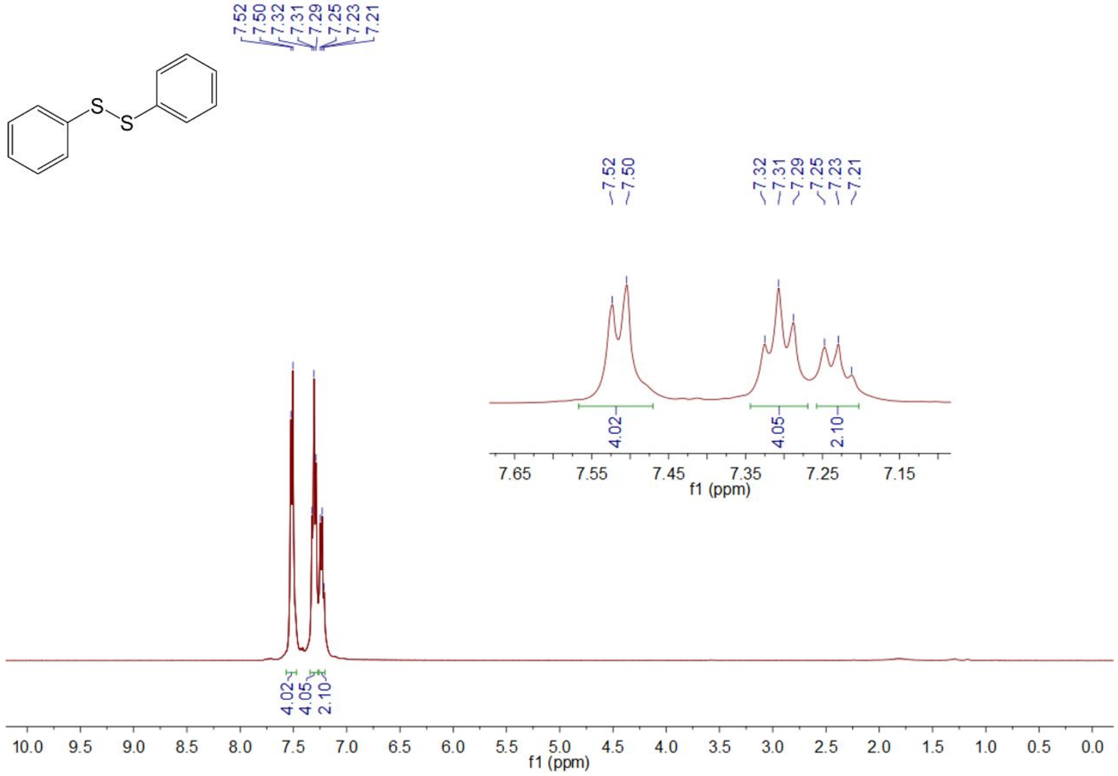

Spectrum S1. ^{1}H NMR spectra of 1,2-diphenyldisulfane in $CDCl_3$.

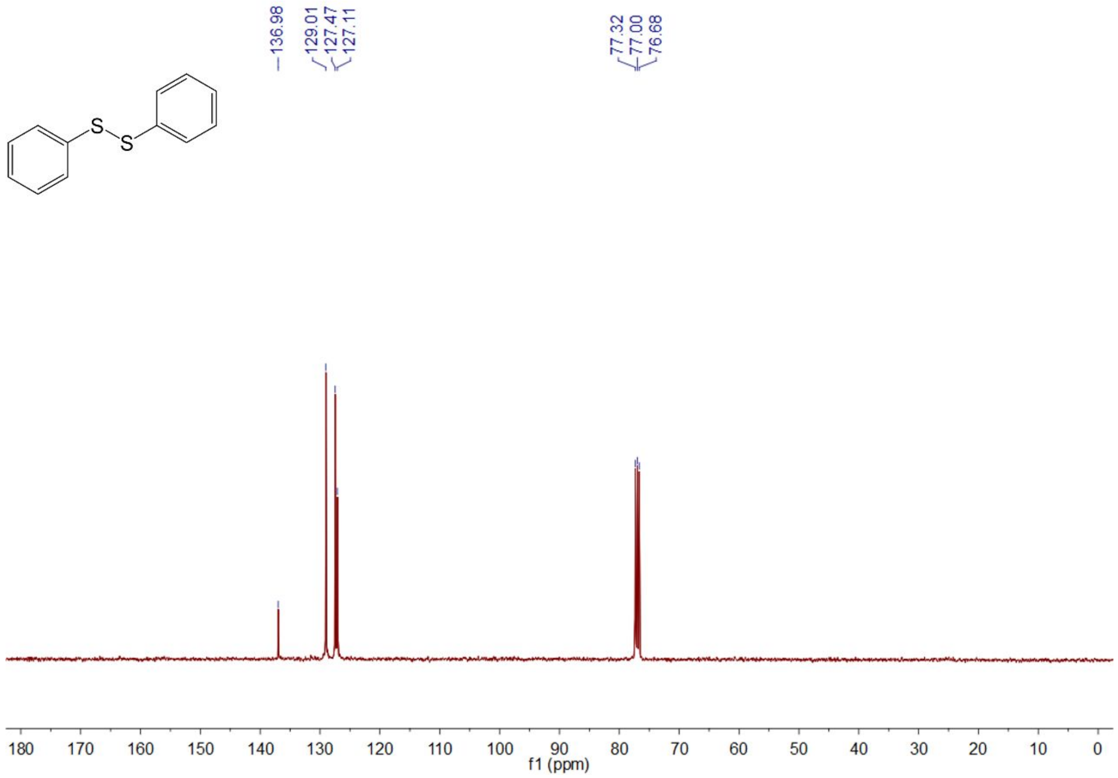

Spectrum S2. ^{13}C NMR spectra of 1,2-diphenyldisulfane in $CDCl_3$.

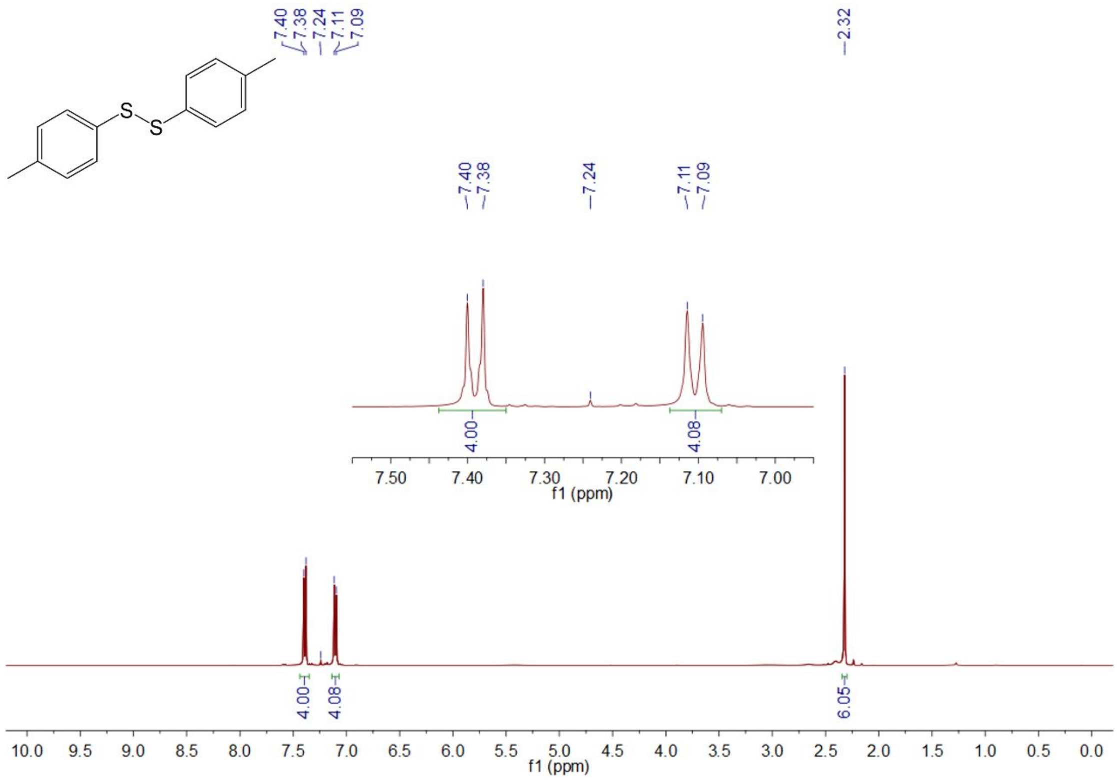

Spectrum S3. ^{1}H NMR spectra of 1,2-di-*p*-tolyldisulfane in $CDCl_3$.

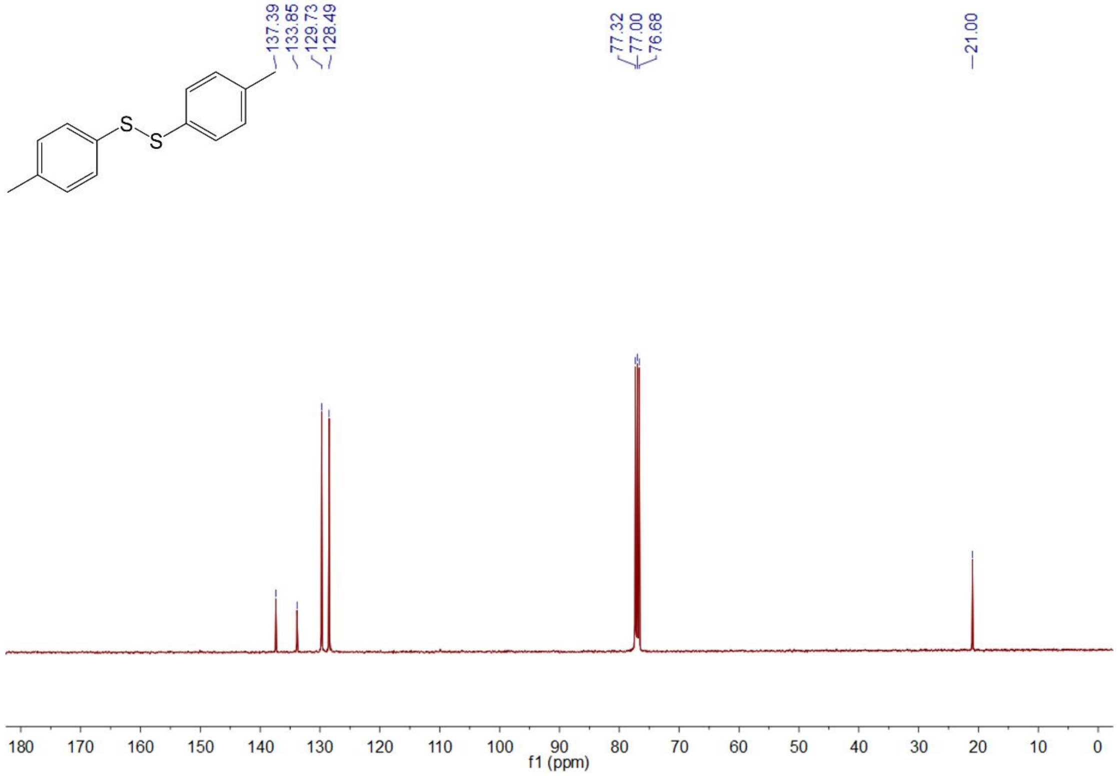

Spectrum S4. ^{13}C NMR spectra of 1,2-di-*p*-tolyldisulfane in $CDCl_3$.

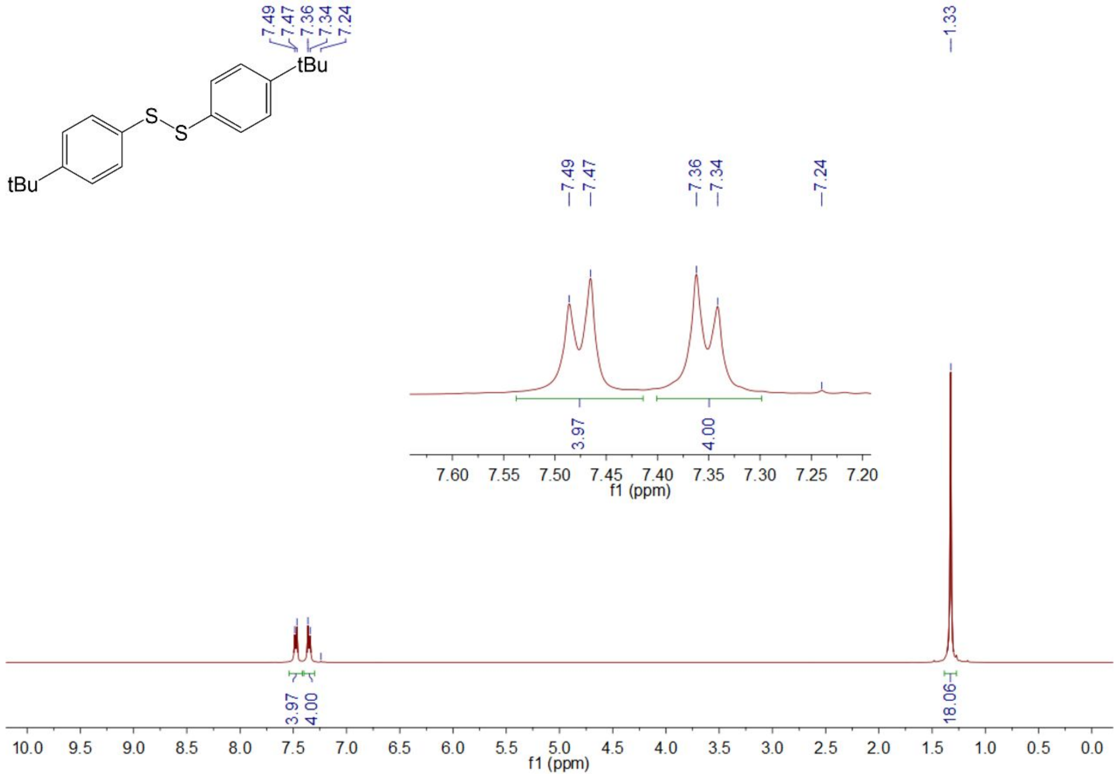

Spectrum S5. ^{1}H NMR spectra of 1,2-bis(4-(tert-butyl)phenyl)disulfane in $CDCl_3$.

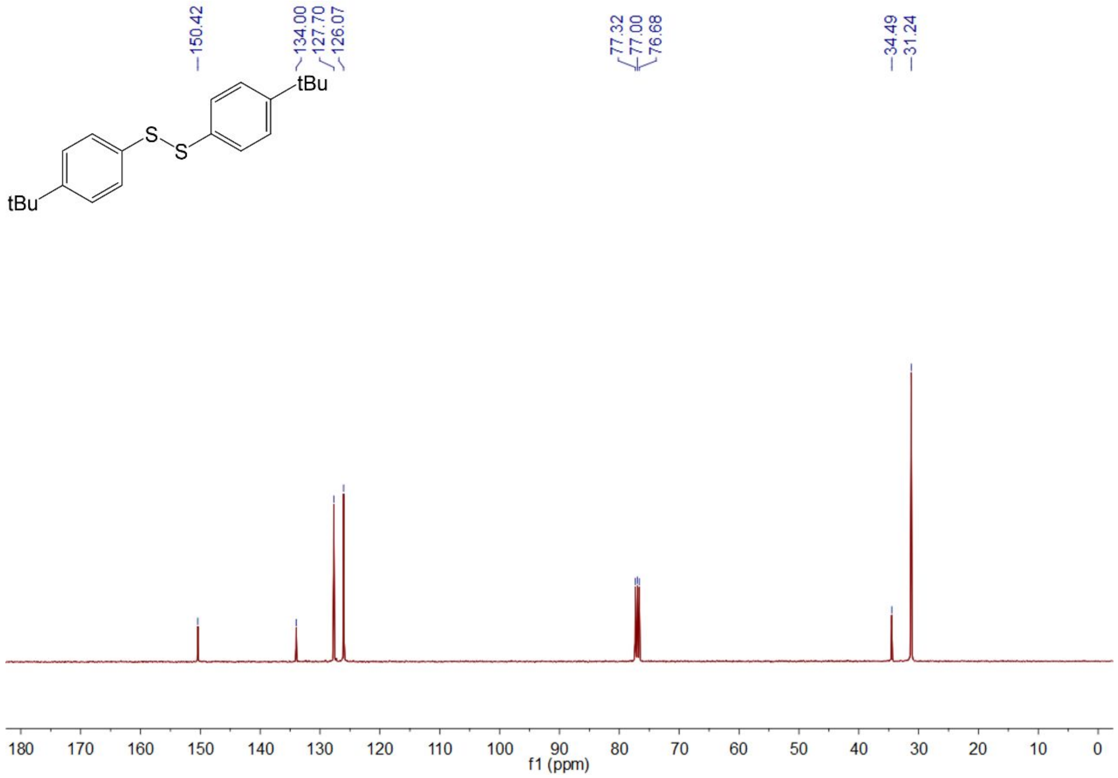

Spectrum S6. ^{13}C NMR spectra of 1,2-bis(4-(tert-butyl)phenyl)disulfane in $CDCl_3$.

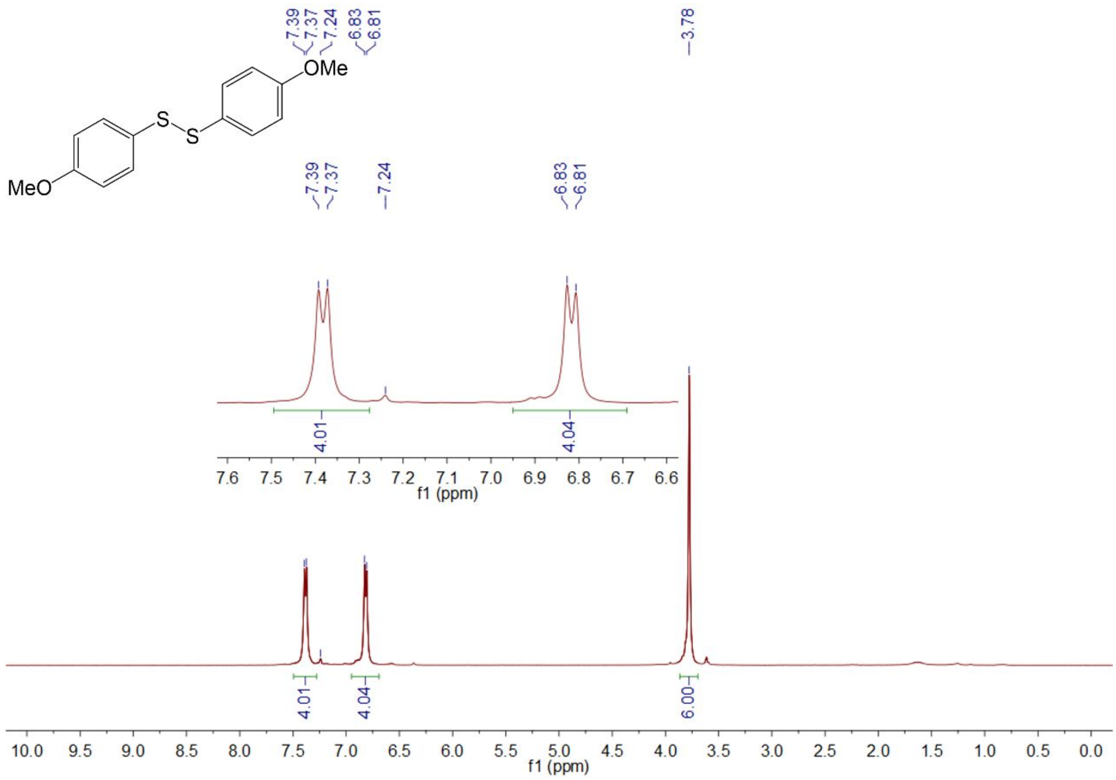

Spectrum S7. ^{1}H NMR spectra of 1,2-bis(4-methoxyphenyl)disulfane in $CDCl_3$.

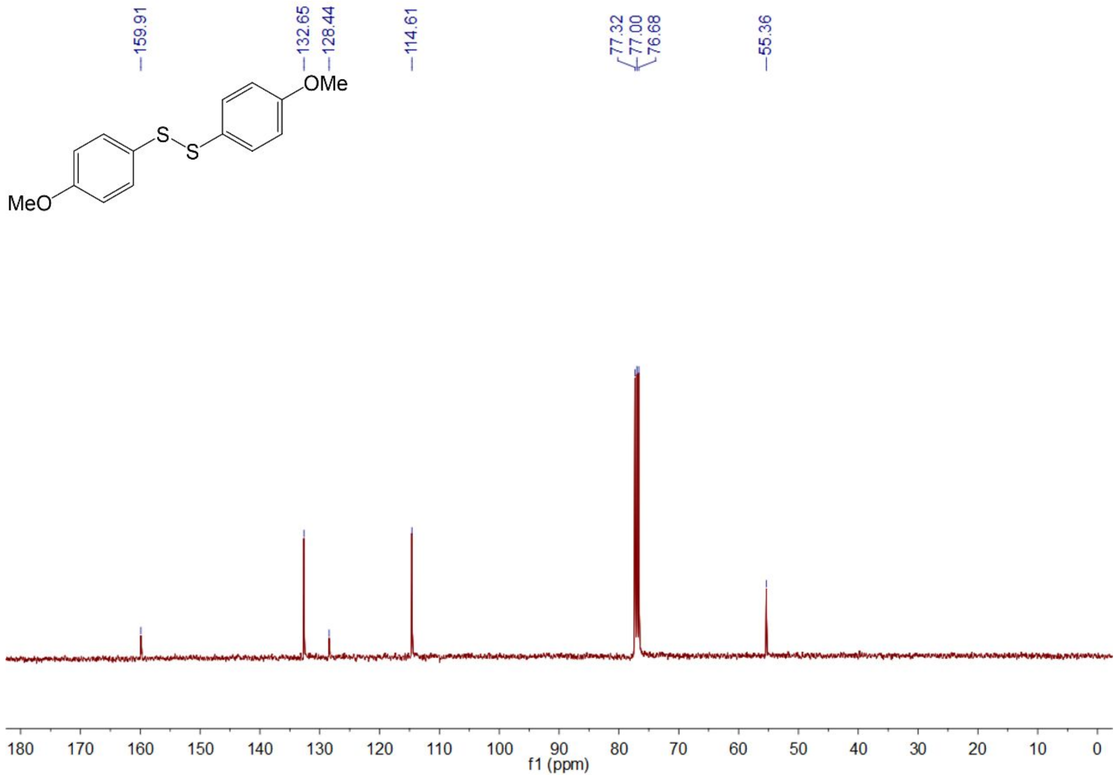

Spectrum S8. ^{13}C NMR spectra of 1,2-bis(4-methoxyphenyl)disulfane in $CDCl_3$.

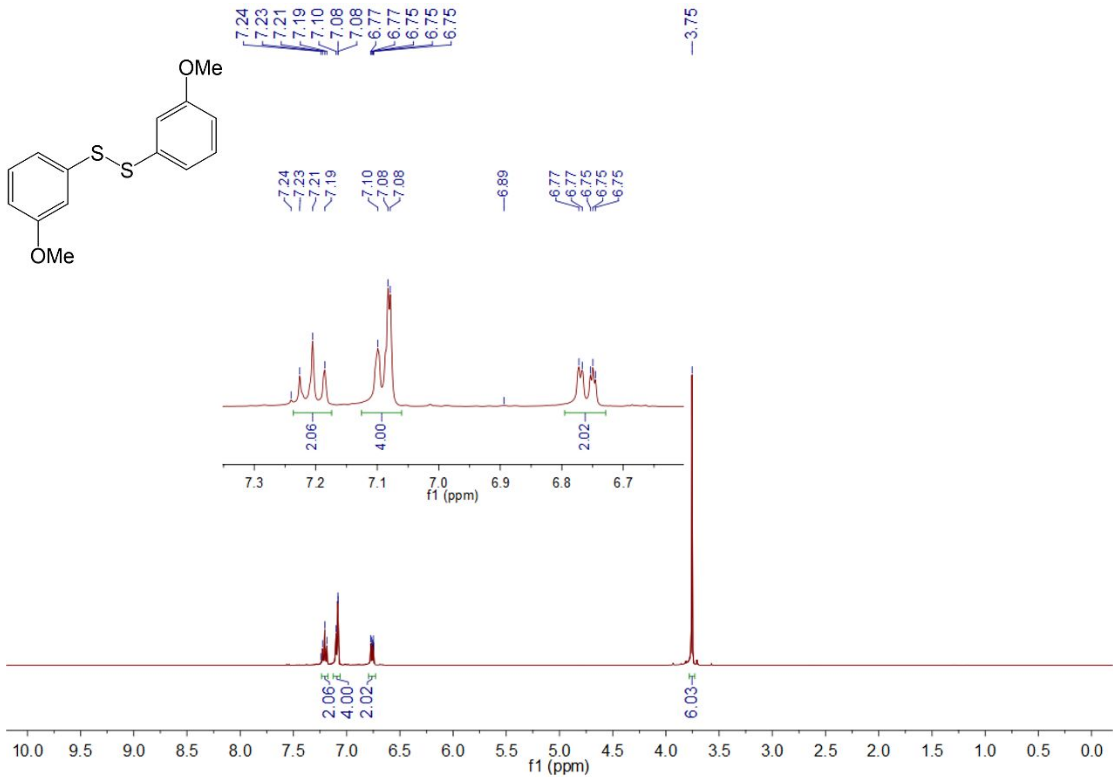

Spectrum S9. ^{1}H NMR spectra of 1,2-bis(3-methoxyphenyl)disulfane in $CDCl_3$.

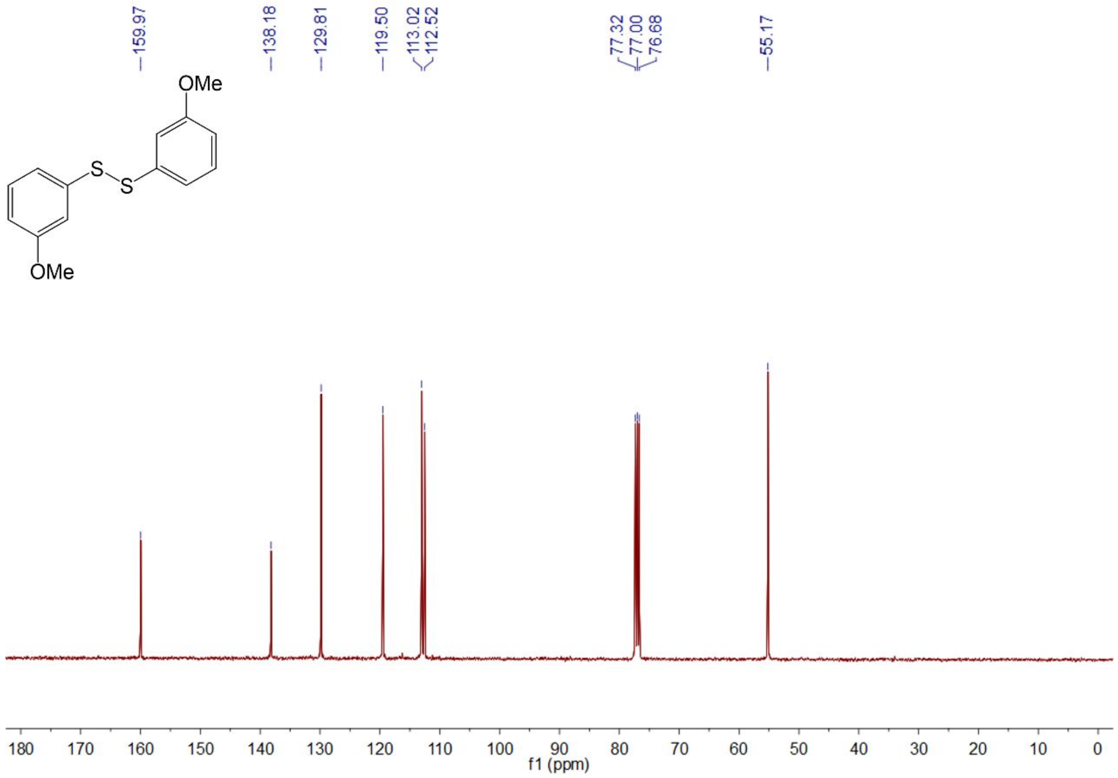

Spectrum S10. ^{13}C NMR spectra of 1,2-bis(3-methoxyphenyl)disulfane in $CDCl_3$.

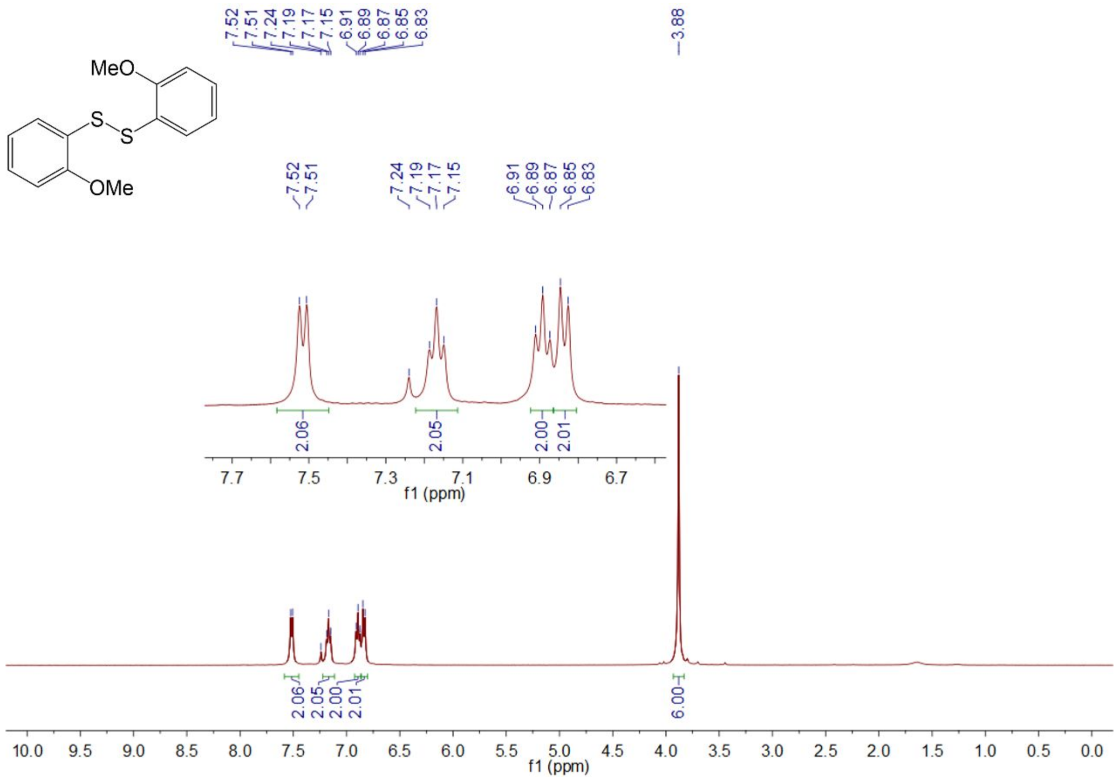

Spectrum S11. ^{1}H NMR spectra of 1,2-bis(2-methoxyphenyl)disulfane in $CDCl_3$.

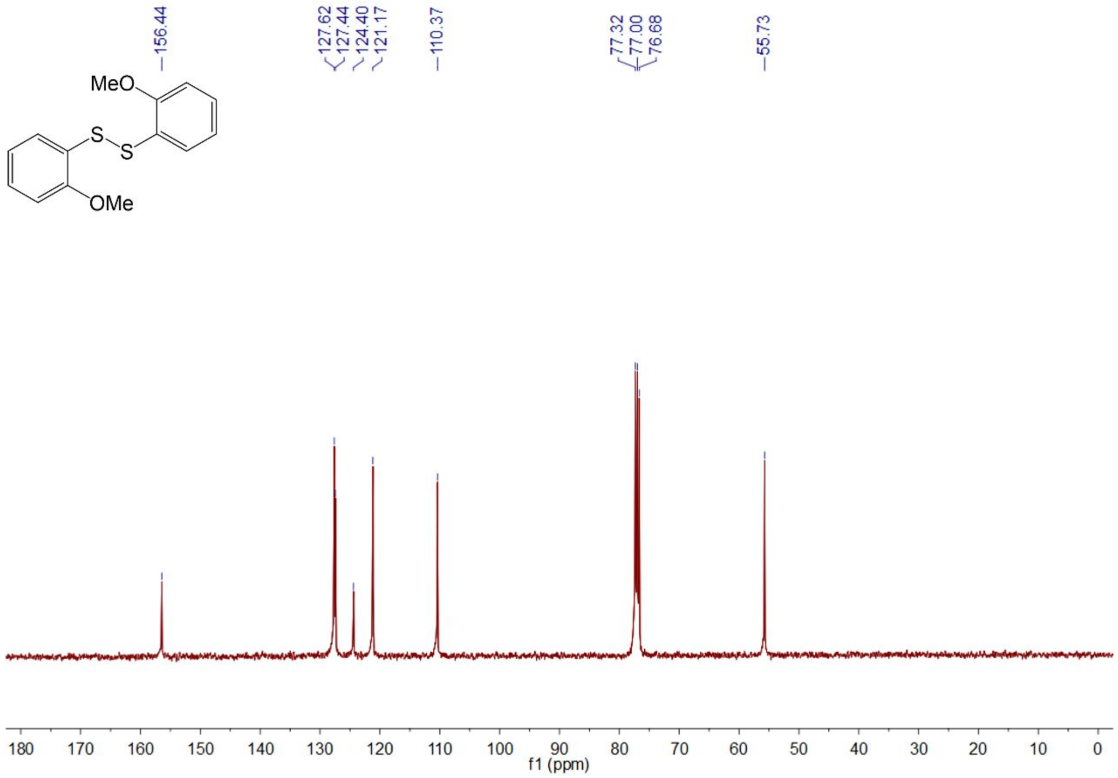

Spectrum S12. ^{13}C NMR spectra of 1,2-bis(2-methoxyphenyl)disulfane in $CDCl_3$.

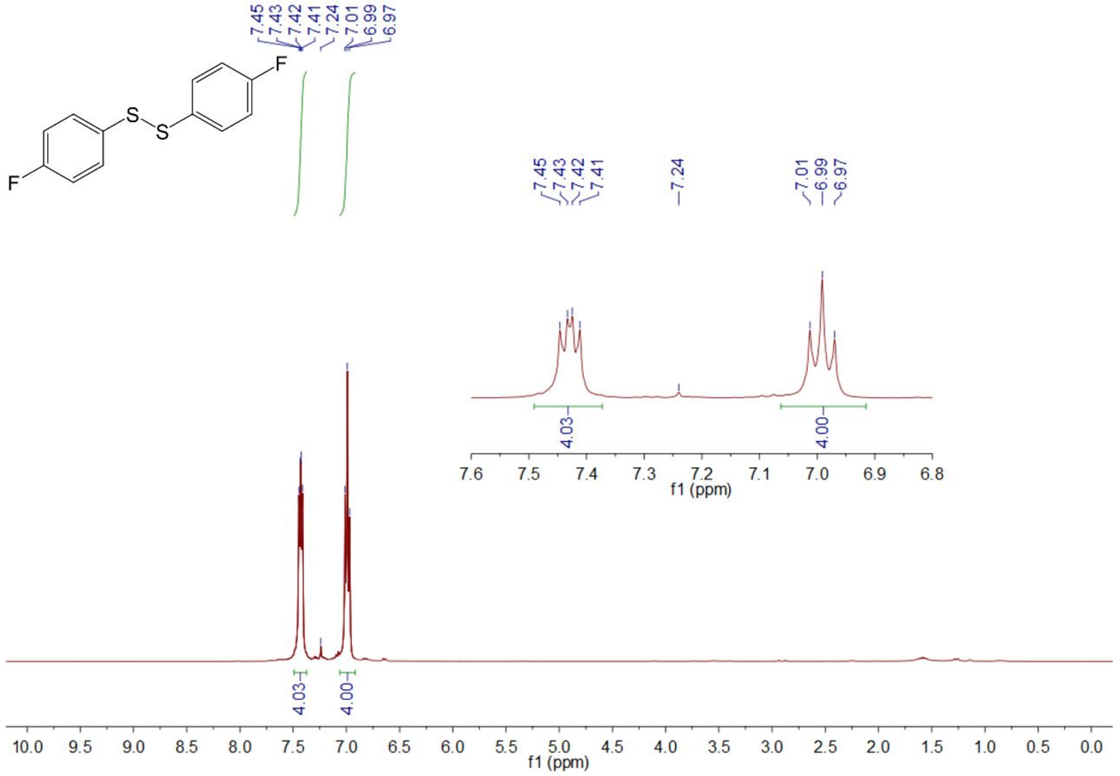

Spectrum S13. ^{1}H NMR spectra of 1,2-bis(4-fluorophenyl)disulfane in $CDCl_3$.

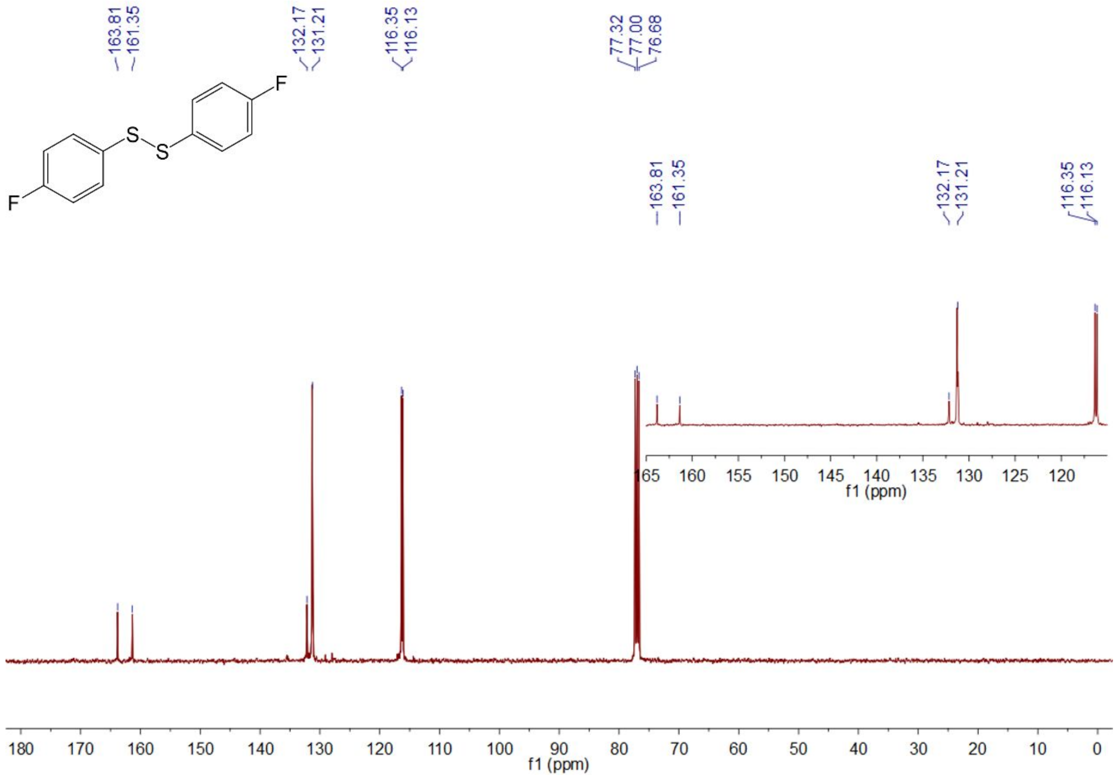

Spectrum S14. ^{13}C NMR spectra of 1,2-bis(4-fluorophenyl)disulfane in $CDCl_3$.

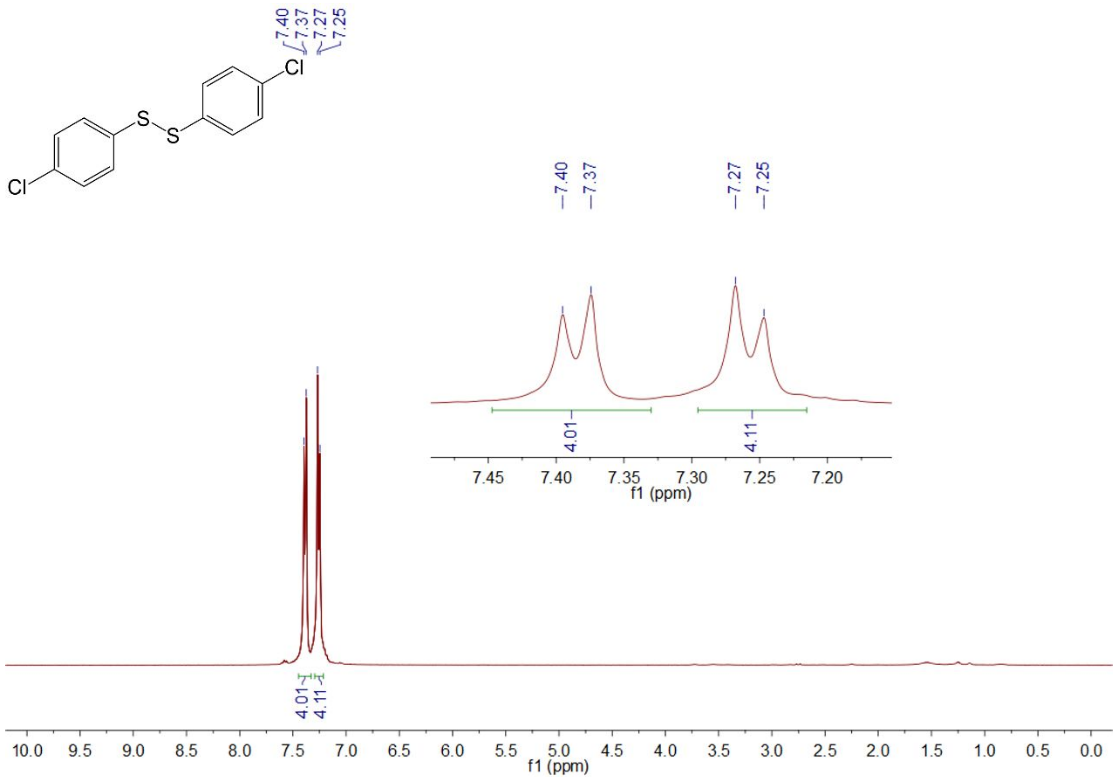

Spectrum S15. ^{1}H NMR spectra of 1,2-bis(4-chlorophenyl)disulfane in $CDCl_3$.

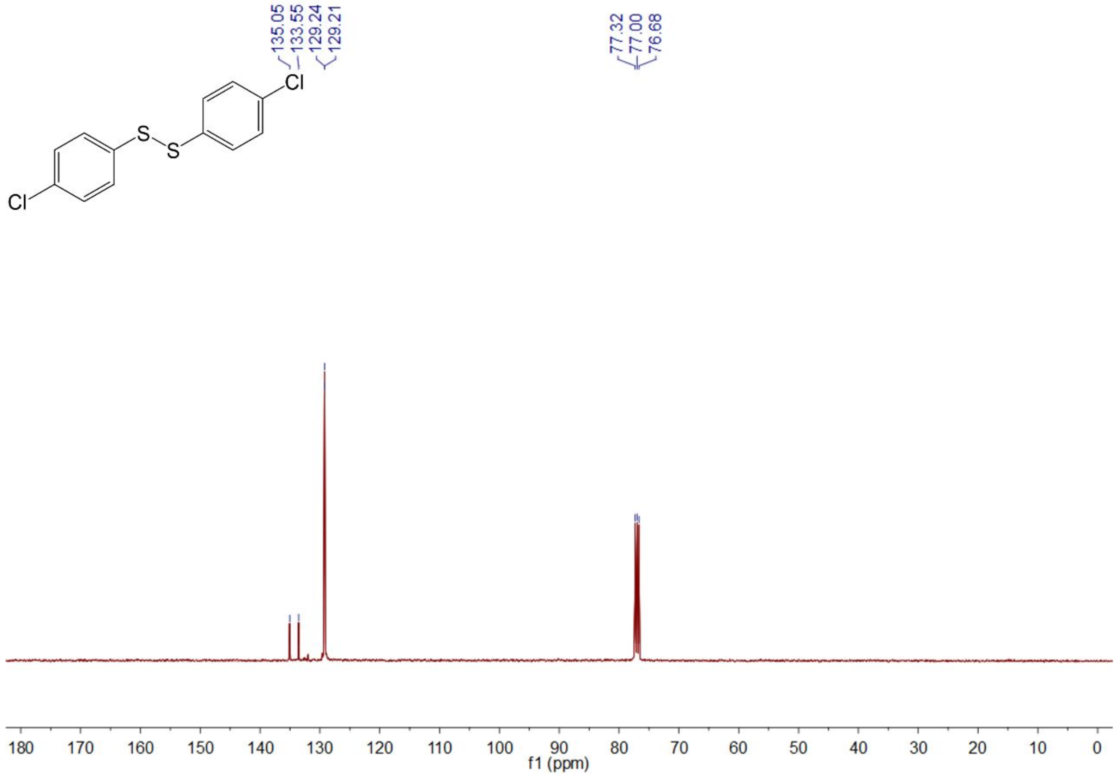

Spectrum S16. ^{13}C NMR spectra of 1,2-bis(4-chlorophenyl)disulfane in $CDCl_3$.

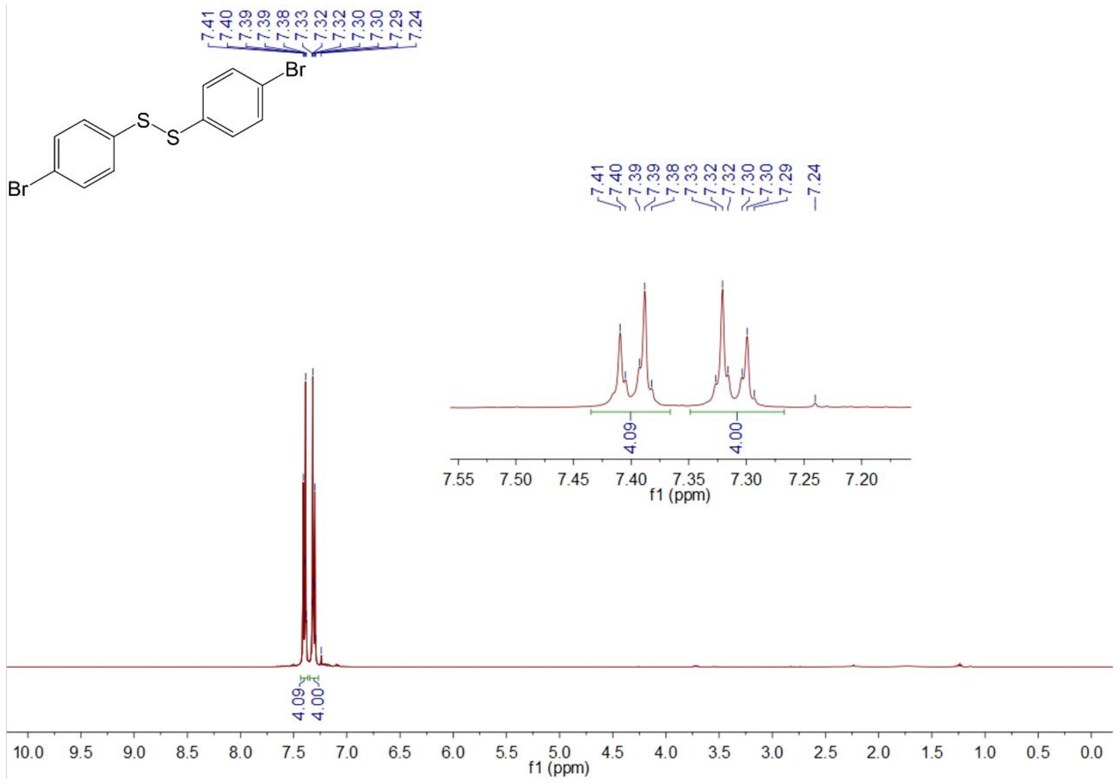

Spectrum S17. ^{1}H NMR spectra of 1,2-bis(4-bromophenyl)disulfane in $CDCl_3$.

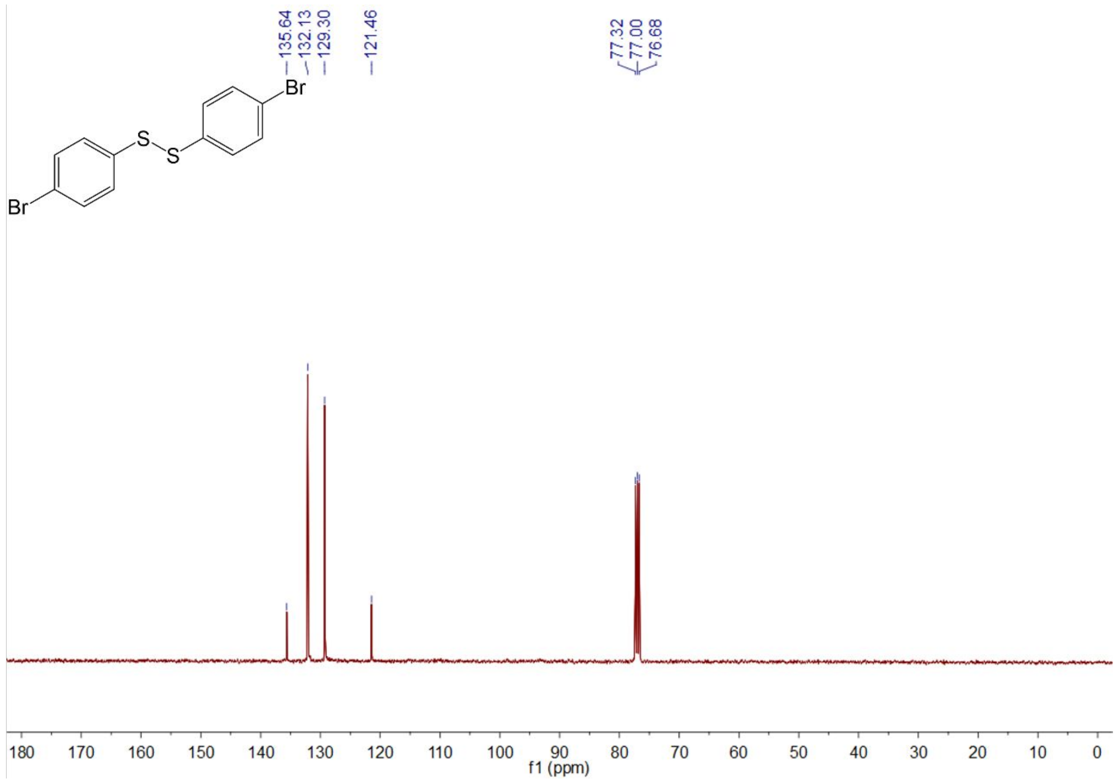

Spectrum S18. ^{13}C NMR spectra of 1,2-bis(4-bromophenyl)disulfane in $CDCl_3$.

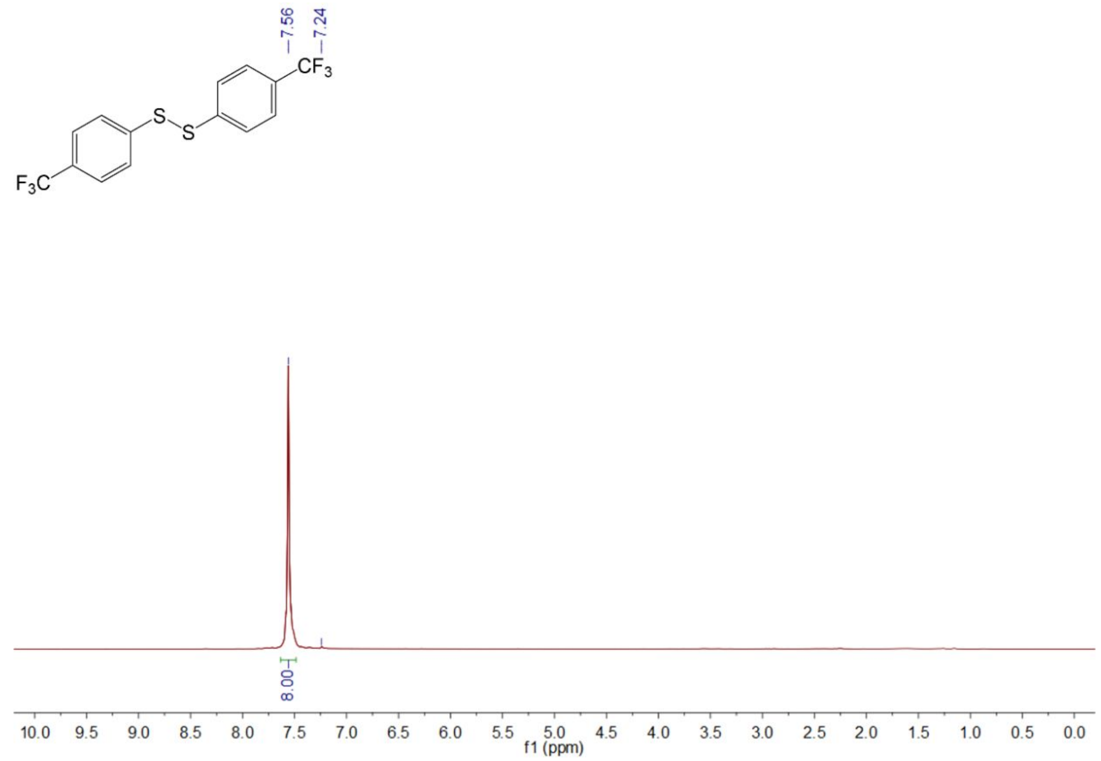

Spectrum S19. ^{1}H NMR spectra of 1,2-bis(4-(trifluoromethyl)phenyl)disulfane in $CDCl_3$.

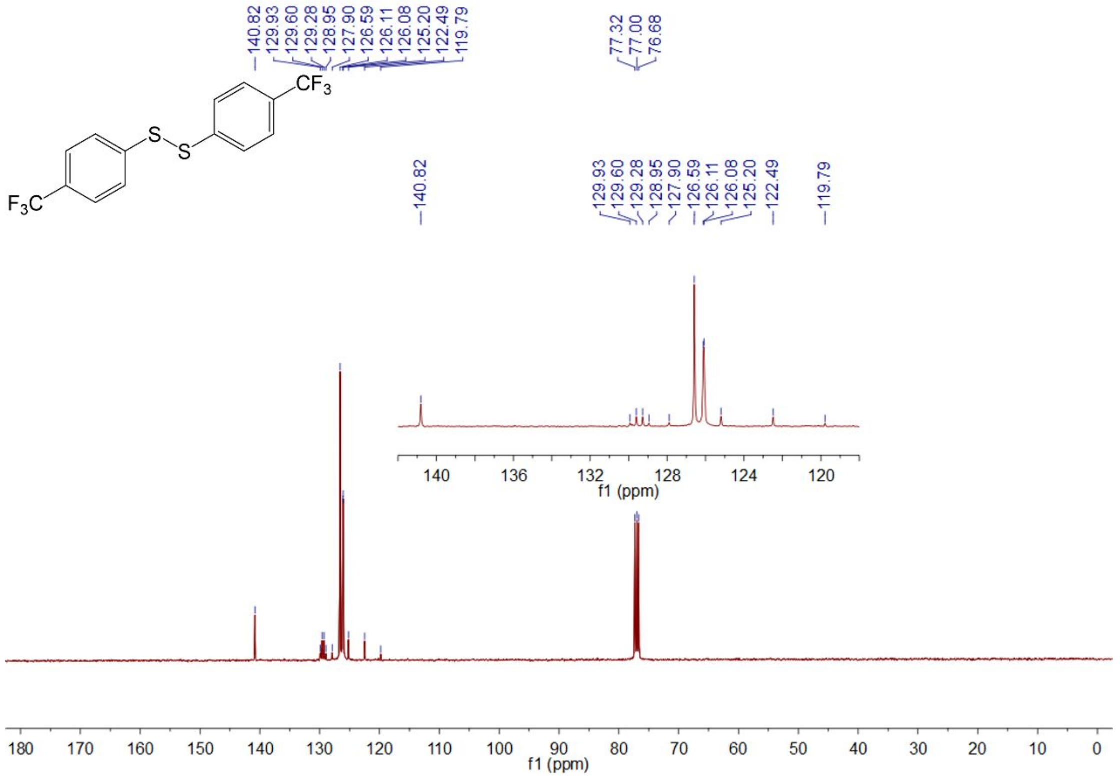

Spectrum S20. ^{13}C NMR spectra of 1,2-bis(4-(trifluoromethyl)phenyl)disulfane in $CDCl_3$.

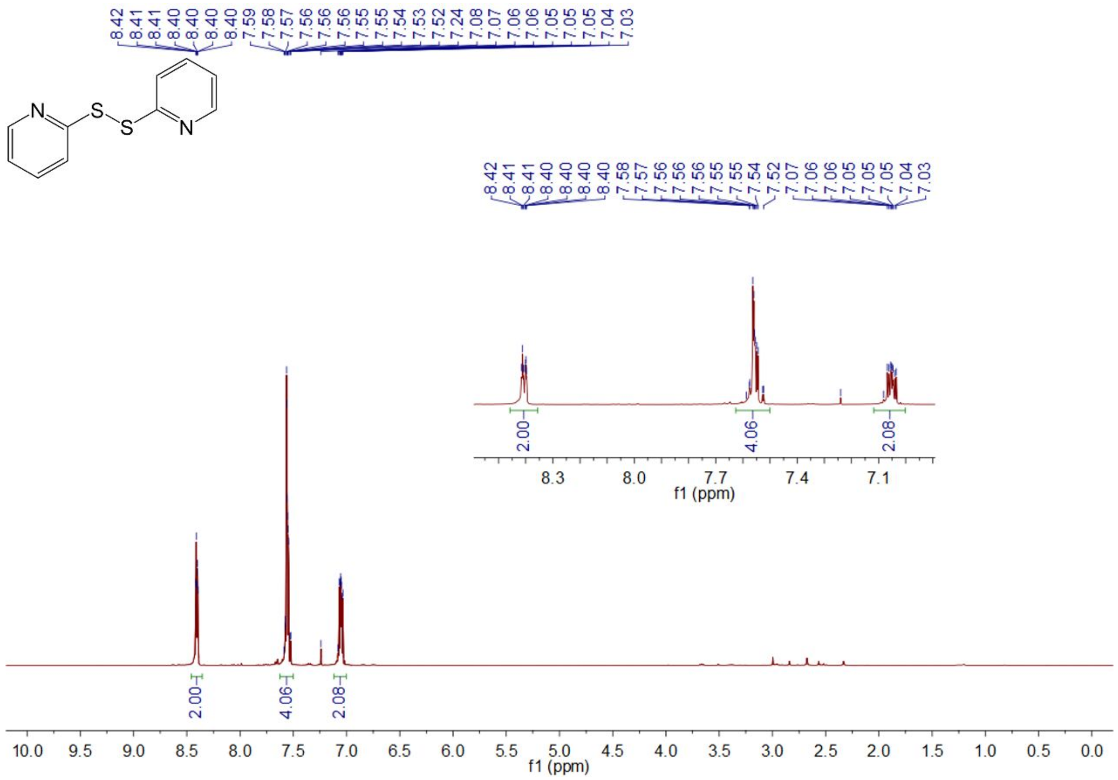

Spectrum S21. ^{1}H NMR spectra of 1,2-di(pyridin-2-yl)disulfane in $CDCl_3$.

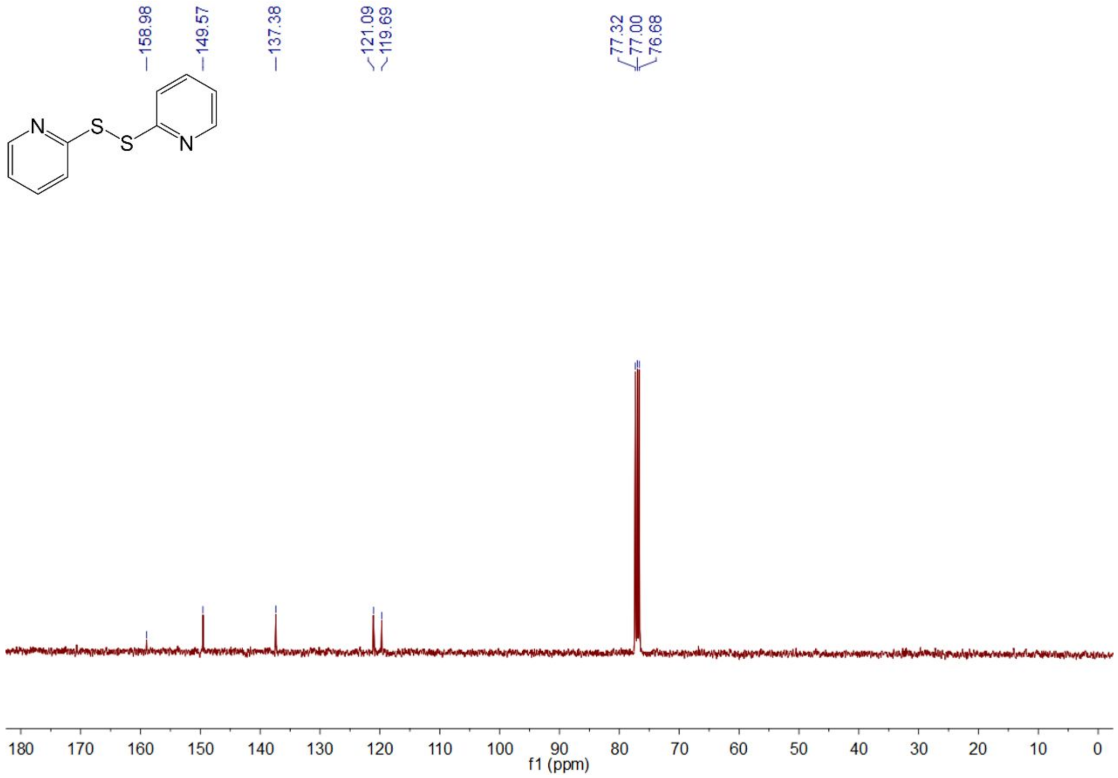

Spectrum S22. ^{13}C NMR spectra of 1,2-di(pyridin-2-yl)disulfane in $CDCl_3$.

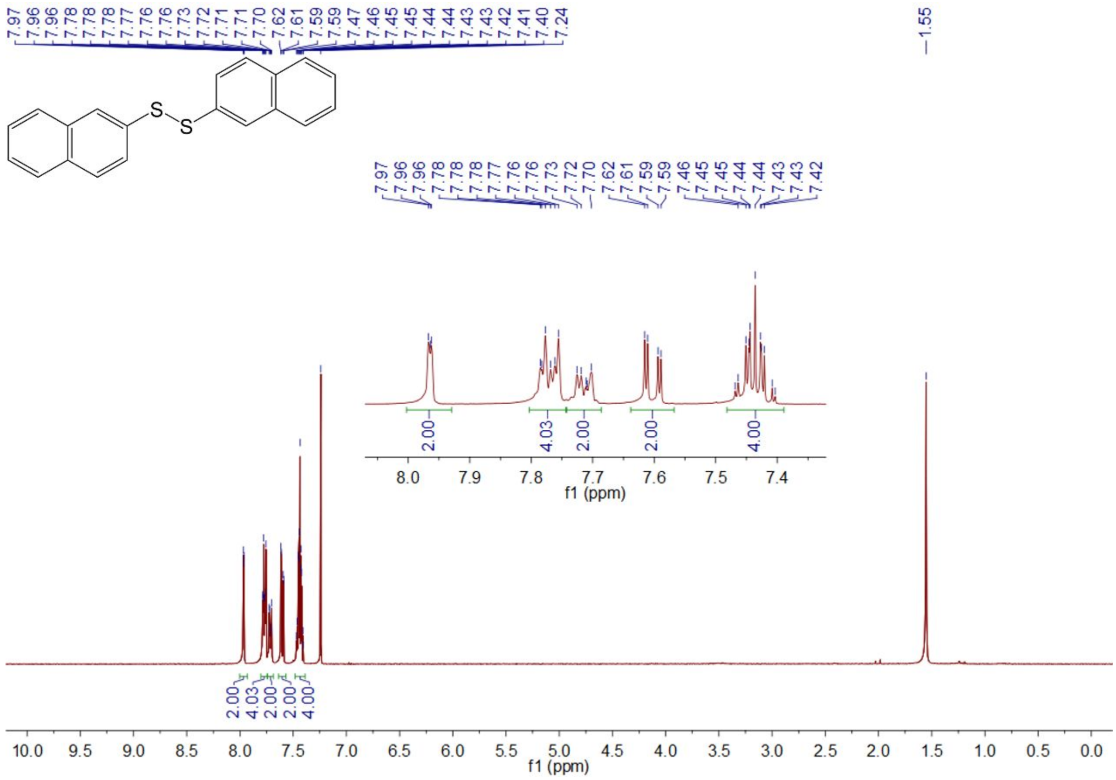

Spectrum S23. ^{1}H NMR spectra of 1,2-di(naphthalen-2-yl)disulfane in $CDCl_3$.

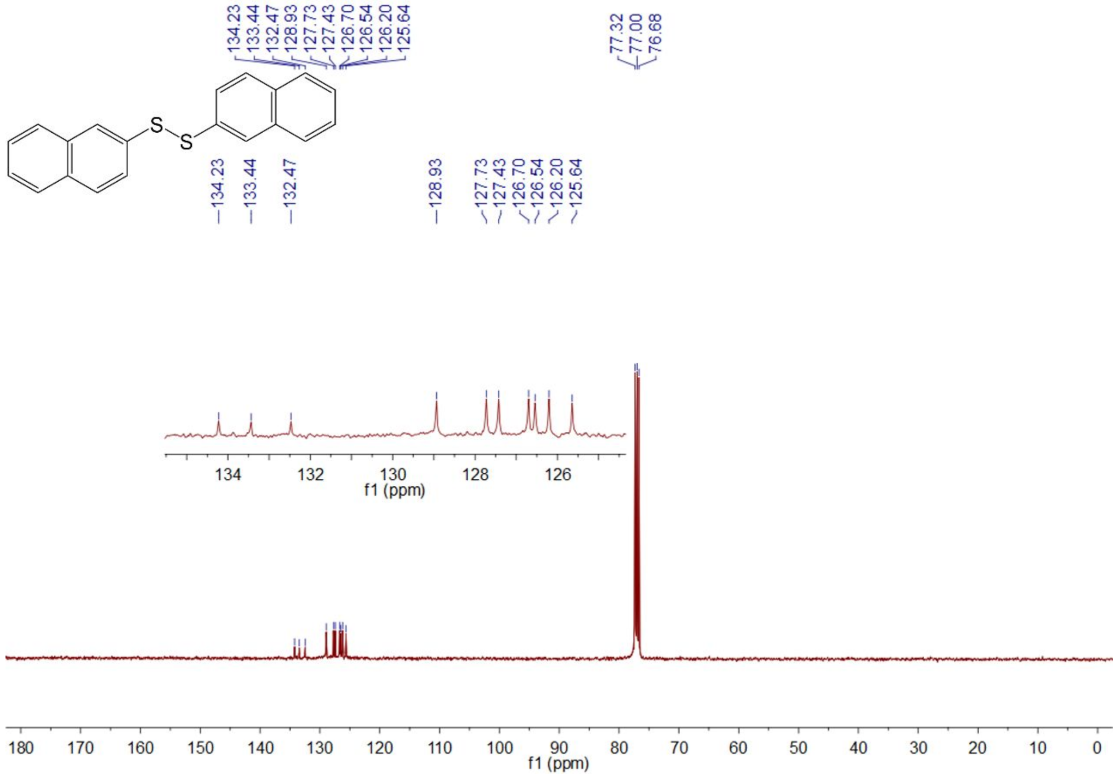

Spectrum S24. ^{13}C NMR spectra of 1,2-di(naphthalen-2-yl)disulfane in $CDCl_3$.
